# Supplementary material for: The value of supportive care: A systematic review of cost-effectiveness of non-pharmacological interventions for dementia
Source: PLoS One. 2023 May 12;18(5):e0285305. doi: 10.1371/journal.pone.0285305 (PMC10180718; doi:10.1371/journal.pone.0285305)
Supplement: S1 File — (DOCX) [file pone.0285305.s003.docx]

**S1 File**

**Electronic Search Strategy**

A systematic literature review was performed between February 2019 and December 2021 (time of revision). We considered studies published through December 2021 with no lower date limit.

The following are the electronic search terms we used in the databases MEDLINE (PubMed), CDSR (Cochrane Database of Systematic Reviews), CENTRAL (Cochrane Central Register of Controlled Trials), Embase and PsycINFO:

*(dementia OR alzheimer* OR cognitive) AND ('cost-effectiveness'/exp OR 'cost-analysis'/exp OR 'cost-utility'/exp) AND ('non-pharmacological'/exp OR ‘psychosocial*’/exp OR 'drug-free'/exp)*

The search strategy can be described more clearly as follows. The numbered items below were combined using the AND Boolean logic operator, whereas items marked by a letter were combined using the OR Boolean logic operator. The asterisk after some terms (*) indicates unlimited truncation. Items marked with a ★ were “exploded”, i.e. we asked the database to also capture all narrower terms associated with the broader concept.

1. Search for terms in the title or abstract related to any form of dementia:
   1. dementia OR
   2. alzheimer* (to capture Alzheimer, Alzheimer’s, etc) OR
   3. cognitive (to capture “cognitive impairment” or related terms)
2. AND search for terms in the title or abstract indicating a value for money appraisal:
   1. cost-effectiveness ★ OR
   2. cost-analysis ★ OR
   3. cost-utility ★
3. AND search for terms in the title or abstract that limit the focus on non-pharmacological interventions:
   1. non-pharmacological ★ OR
   2. psychosocial* ★ OR
   3. drug-free ★
