# Supplementary material for: The value of supportive care: A systematic review of cost-effectiveness of non-pharmacological interventions for dementia
Source: PLoS One. 2023 May 12;18(5):e0285305. doi: 10.1371/journal.pone.0285305 (PMC10180718; doi:10.1371/journal.pone.0285305)
Supplement: S4 Table — (DOCX) [file pone.0285305.s007.docx]

**S4 Table.** **Assessment of methodological quality of the studies on interventions primarily aimed at supporting family caregivers**

**Consensus on Health Economic Criteria (CHEC) checklist (*)**

|  | Knapp et al. 2013 [71] | Livingston et al. 2014 [117] | Livingston et al. 2019 [118] | Nichols et al. 2008 [73] | Drummond et al. 1991 [69] | Shaw et al. 2020 [76] | Gaugler et al. 2003 [131] | Joling et al. 2013 [72] | Wilson et al. 2009 [132] |
| --- | --- | --- | --- | --- | --- | --- | --- | --- | --- |
| **1. Study population clearly described?** | x | x | x | x | x | x | x | x | x |
| **2. Competing alternatives clearly described?** | x | x | x | x | x | x | x | x | x |
| **3. Well-defined research question in answerable form?** | x | x | x | x |  | x | x | x | x |
| **4. Economic study design appropriate to stated objective?** | x | x | x | x | x | x | x | x | x |
| **5. Chosen time horizon appropriate to include relevant costs and consequences?** |  | x | x | x | x |  |  | x | x |
| **6. Actual perspective chosen appropriate?** | x |  | x | x | x |  | x | x | x |
| **7. All important and relevant costs for each alternative identified?** | x | x | x | x | x | x | x | x | x |
| **8. All costs measured appropriately in physical units?** | x | x | x | x | x | x | x | x | x |
| **9. Costs valued appropriately?** | x | x | x | x | x |  |  | x | x |
| **10. All important and relevant outcomes for each alternative identified?** | x | x | x | x | x | x | x | x | x |
| **11. All outcomes measured appropriately?** | x | x | x | x | x | x | x | x | x |
| **12. Outcomes valued appropriately?** | x | x | x | x | x | x |  | x | x |
| **13. Incremental analysis of costs and outcomes of alternatives performed?** | x | x |  | x | x | x |  | x | x |
| **14. All future costs and outcomes discounted appropriately?** | NA | x | x | NA | NA | NA | x | NA | x |
| **15. All important variables, whose values are uncertain, appropriately subjected to sensitivity analysis?** | x | x |  |  |  |  |  | x | x |
| **16. Do conclusions follow from the data reported?** | x | x | x | x | x | x | x | x | x |
| **17. Study discusses generalizability of results to other settings and patient/client groups?** | x |  | x | x |  |  |  | x | x |
| **18. Article indicates that there is no potential conflict of interest of study researcher(s) and funder(s)?** | x | x | x | x |  | x | x | x | x |
| **19. Ethical and distributional issues discussed appropriately?** | x |  |  |  |  |  |  |  | x |
| **Quality Score** | 17 | 16 | 16 | 16 | 13 | 12 | 12 | 17 | 19 |
| **Quality Level (H=High; M=Medium; L=Low)** | H | M | M | M | L | L | L | H | H |

(*) Evers S, Goossens M, de Vet H, van Tulder M, Ament A. Criteria list for assessment of methodological quality of economic evaluations: Consensus on Health Economic Criteria. Int J Technol Assess Health Care [Internet]. 2005 Apr; 21(2):240–5. doi: 10.1017/s0266462305050324

(NA = Not Applicable)
