# Supplementary material for: The value of supportive care: A systematic review of cost-effectiveness of non-pharmacological interventions for dementia
Source: PLoS One. 2023 May 12;18(5):e0285305. doi: 10.1371/journal.pone.0285305 (PMC10180718; doi:10.1371/journal.pone.0285305)
Supplement: Attachment — Submitted filename: PONE-D-21-04004.pdf [file pone.0285305.s009.pdf]

PLOS ONE

Less drugs and more care: A systematic review of cost-effectiveness of supportive care interventions for dementia

--Manuscript Draft--

**Manuscript Number:**	PONE-D-21-04004
**Article Type:**	Research Article
**Full Title:**	Less drugs and more care: A systematic review of cost-effectiveness of supportive care interventions for dementia
**Short Title:**	Cost-effectiveness of supportive care interventions for dementia
**Corresponding Author:**	Vincenzo Rebba<br>University of Padova: Universita degli Studi di Padova<br>Padova, Italy ITALY
**Keywords:**	dementia; non-pharmacological interventions; supportive care; cost-effectiveness
**Abstract:**	Background: Almost 44 million people are currently living with dementia worldwide. This number is set to increase threefold by 2050, posing a serious threat to the sustainability of healthcare systems. Overuse of antipsychotic drugs for the management of the symptoms of dementia carries negative consequences for patients while also increasing the health expenditures for the society. Supportive care interventions could be considered a safer and potentially cost-saving option. In this paper we provide a systematic review of the existing evidence regarding the cost-effectiveness and cost-utility of SCIs that are targeted towards persons living with dementia and their caregivers.<br>Methods: A systematic literature review was performed between February 2019 and March 2020 through searches of major healthcare electronic databases, including the Cochrane Library and PubMed (MEDLINE). The search strategy was based on PRISMA recommendations. We distinguished between five categories of supportive care strategies: cognitive therapies, physical activity, indirect strategies (organisational and environmental changes), interventions primarily targeted towards caregivers, and multicomponent interventions.<br>Results: Of the 3,221 articles retrieved, 33 met the inclusion criteria. These studies analysed 29 supportive care programmes located at different stages of the care pathway for dementia. Ten studies provided evidence of high cost-effectiveness for seven interventions: a multicomponent intervention targeted towards nursing home residents; two cognitive stimulation and occupational programmes for community-dwelling persons with dementia; two indirect interventions; two interventions aimed at caregivers of community-dwelling persons with dementia.<br>Conclusion: We find that the most promising supportive care strategies in terms of cost-effectiveness are multicomponent interventions targeted towards nursing home residents, some forms of tailored occupational therapy and home care support services, together with some forms of psychosocial intervention for informal caregivers of community-dwelling persons with dementia. Our results suggest that the adoption of effective supportive care interventions may increase the economic sustainability of dementia care.
**Order of Authors:**	Angelica Guzzon
	Vincenzo Rebba
	Omar Paccagnella
	Michela Rigon
	Giovanni Boniolo
**Additional Information:**	
**Question**	**Response**
**Financial Disclosure**	Funding from the University of Padua and Fondazione OIC is gratefully acknowledged.

Powered by Editorial Manager® and ProduXion Manager® from Aries Systems Corporation

Enter a financial disclosure statement that describes the sources of funding for the work included in this submission. Review the submission guidelines for detailed requirements. View published research articles from *PLOS ONE* for specific examples.<br><br>This statement is required for submission and **will appear in the published article** if the submission is accepted. Please make sure it is accurate.<br><br>**Unfunded studies**<br>Enter: *The author(s) received no specific funding for this work.*<br><br>**Funded studies**<br>Enter a statement with the following details:<br>• Initials of the authors who received each award<br>• Grant numbers awarded to each author<br>• The full name of each funder<br>• URL of each funder website<br>• Did the sponsors or funders play any role in the study design, data collection and analysis, decision to publish, or preparation of the manuscript?<br>• **NO** - Include this sentence at the end of your statement: *The funders had no role in study design, data collection and analysis, decision to publish, or preparation of the manuscript.*<br>• **YES** - Specify the role(s) played.<br><br>* typeset	
**Competing Interests**<br><br>Use the instructions below to enter a competing interest statement for this submission. On behalf of all authors, disclose any competing interests that could be perceived to bias this work—acknowledging all financial support and any other relevant financial or non-financial competing interests.<br><br>This statement is required for submission and **will appear in the published article** if the submission is accepted. Please make	The authors have declared that no competing interests exist.

sure it is accurate and that any funding sources listed in your Funding Information later in the submission form are also declared in your Financial Disclosure statement.<br><br>View published research articles from *PLOS ONE* for specific examples.<br><br>**NO authors have competing interests**<br><br>Enter: *The authors have declared that no competing interests exist.*<br><br>**Authors with competing interests**<br><br>Enter competing interest details beginning with this statement:<br><br>*I have read the journal's policy and the authors of this manuscript have the following competing interests: [insert competing interests here]*<br><br>* typeset	
**Ethics Statement**<br><br>Enter an ethics statement for this submission. This statement is required if the study involved:<br><br>• Human participants<br>• Human specimens or tissue<br>• Vertebrate animals or cephalopods<br>• Vertebrate embryos or tissues<br>• Field research<br><br>Write "N/A" if the submission does not require an ethics statement.<br><br>General guidance is provided below. Consult the submission guidelines for detailed instructions. **Make sure that all information entered here is included in the Methods section of the manuscript.**	N/A

**Format for specific study types**<br><br>**Human Subject Research (involving human participants and/or tissue)**<br>• Give the name of the institutional review board or ethics committee that approved the study<br>• Include the approval number and/or a statement indicating approval of this research<br>• Indicate the form of consent obtained (written/oral) or the reason that consent was not obtained (e.g. the data were analyzed anonymously)<br><br>**Animal Research (involving vertebrate animals, embryos or tissues)**<br>• Provide the name of the Institutional Animal Care and Use Committee (IACUC) or other relevant ethics board that reviewed the study protocol, and indicate whether they approved this research or granted a formal waiver of ethical approval<br>• Include an approval number if one was obtained<br>• If the study involved *non-human primates*, add *additional details* about animal welfare and steps taken to ameliorate suffering<br>• If anesthesia, euthanasia, or any kind of animal sacrifice is part of the study, include briefly which substances and/or methods were applied<br><br>**Field Research**<br><br>Include the following details if this study involves the collection of plant, animal, or other materials from a natural setting:<br>• Field permit number<br>• Name of the institution or relevant body that granted permission	
**Data Availability**<br><br>Authors are required to make all data underlying the findings described fully available, without restriction, and from the time of publication. PLOS allows rare exceptions to address legal and ethical concerns. See the PLOS Data Policy and FAQ for detailed information.	Yes - all data are fully available without restriction

A Data Availability Statement describing where the data can be found is required at submission. Your answers to this question constitute the Data Availability Statement and **will be published in the article**, if accepted.<br><br>**Important:** Stating 'data available on request from the author' is not sufficient. If your data are only available upon request, select 'No' for the first question and explain your exceptional situation in the text box.<br><br>Do the authors confirm that all data underlying the findings described in their manuscript are fully available without restriction?	
**Describe where the data may be found in full sentences. If you are copying our sample text, replace any instances of XXX with the appropriate details.**<br><br>• If the data are **held or will be held in a public repository**, include URLs, accession numbers or DOIs. If this information will only be available after acceptance, indicate this by ticking the box below. For example: *All XXX files are available from the XXX database (accession number(s) XXX, XXX.)*.<br>• If the data are all contained **within the manuscript and/or Supporting Information files**, enter the following: *All relevant data are within the manuscript and its Supporting Information files.*<br>• If neither of these applies but you are able to provide **details of access elsewhere**, with or without limitations, please do so. For example:<br><br>*Data cannot be shared publicly because of [XXX]. Data are available from the XXX Institutional Data Access / Ethics Committee (contact via XXX) for researchers who meet the criteria for access to confidential data.*<br><br>*The data underlying the results presented in the study are available from (include the name of the third party*	All relevant data are within the manuscript and its Supporting Information files.

Powered by Editorial Manager® and ProduXion Manager® from Aries Systems Corporation

and contact information or URL).<br>• This text is appropriate if the data are owned by a third party and authors do not have permission to share the data.<br><br>* typeset	
Additional data availability information:	Tick here if the URLs/accession numbers/DOIs will be available only after acceptance of the manuscript for publication so that we can ensure their inclusion before publication.

Powered by Editorial Manager® and ProduXion Manager® from Aries Systems Corporation

Manuscript Click here to access/download;Manuscript;Manuscript Supportive Care for Dementia.docx

Less drugs and more care: A systematic review of cost-effectiveness of supportive care interventions for dementia

Short title: *Cost-effectiveness of supportive care interventions for dementia*

Angelica Guzzon[1,2,3], Vincenzo Rebba[1,2,4*], Omar Paccagnella[1,5], Michela Rigon[1], Giovanni Boniolo[1,6]

[1] Civitas Vitae Research Centre at Fondazione OIC, Padova, Italy
[2] CRIEP (Interuniversity Research Centre on Public Economics), Italy
[3] Department of Economics, Ca' Foscari University of Venice, Italy
[4] Department of Economics and Management "Marco Fanno", University of Padova, Italy
[5] Department of Statistical Sciences, University of Padova, Italy
[6] Department of Neuroscience and Rehabilitation, University of Ferrara, Italy

* Corresponding author: vincenzo.rebba@unipd.it ORCID No. 0000-0003-4685-1890

Authors contribution

Conceptualization: Angelica Guzzon, Vincenzo Rebba, Giovanni Boniolo
Data curation: Angelica Guzzon, Vincenzo Rebba
Formal analysis: Angelica Guzzon
Investigation: Angelica Guzzon, Vincenzo Rebba
Methodology: Angelica Guzzon, Vincenzo Rebba, Omar Paccagnella, Michela Rigon, Giovanni Boniolo
Project Administration: Vincenzo Rebba
Resources: Giovanni Boniolo, Vincenzo Rebba
Supervision: Giovanni Boniolo, Vincenzo Rebba
Validation: Vincenzo Rebba, Omar Paccagnella, Michela Rigon, Giovanni Boniolo
Visualization: Angelica Guzzon, Vincenzo Rebba
Writing – original draft: Angelica Guzzon, Vincenzo Rebba, Omar Paccagnella

Funding information: Funding from the University of Padua and Fondazione OIC is gratefully acknowledged.

Competing interests: The authors have declared that no competing interests exist.

Keywords: dementia; non-pharmacological interventions; supportive care; cost-effectiveness

Abstract

Background: Almost 44 million people are currently living with dementia worldwide. This number is set to increase threefold by 2050, posing a serious threat to the sustainability of healthcare systems. Overuse of antipsychotic drugs for the management of the symptoms of dementia carries negative consequences for patients while also increasing the health expenditures for the society. Supportive care interventions could be considered a safer and potentially cost-saving option. In this paper we provide a systematic review of the existing evidence regarding the cost-effectiveness and cost-utility of SCIs that are targeted towards persons living with dementia and their caregivers.

Methods: A systematic literature review was performed between February 2019 and March 2020 through searches of major healthcare electronic databases, including the Cochrane Library and PubMed (MEDLINE). The search strategy was based on PRISMA recommendations. We distinguished between five categories of supportive care strategies: cognitive therapies, physical activity, indirect strategies (organisational and environmental changes), interventions primarily targeted towards caregivers, and multicomponent interventions.

Results: Of the 3,221 articles retrieved, 33 met the inclusion criteria. These studies analysed 29 supportive care programmes located at different stages of the care pathway for dementia. Ten studies provided evidence of high cost-effectiveness for seven interventions: a multicomponent intervention targeted towards nursing home residents; two cognitive stimulation and occupational programmes for community-dwelling persons with dementia; two indirect interventions; two interventions aimed at caregivers of community-dwelling persons with dementia.

Conclusion: We find that the most promising supportive care strategies in terms of cost-effectiveness are multicomponent interventions targeted towards nursing home residents, some forms of tailored occupational therapy and home care support services, together with some forms of psychosocial intervention for informal caregivers of community-dwelling persons with dementia. Our results suggest that the adoption of effective supportive care interventions may increase the economic sustainability of dementia care.

Introduction

Globally, about 43.8 million people were living with dementia in 2016 [1], and this number is projected to triple by 2050 [2]. The substantial increase in the prevalence of dementia, mostly driven by demographic trends, poses significant challenges to health and social care systems, especially in terms of economic sustainability. The scale of the problem becomes even greater when we take into account the indirect costs related to caregivers of persons with dementia (PwDs). Caregiving exacts a toll on caregivers' mental, emotional, physical, and financial health [3]; caregivers are twice as likely to suffer from depression [4], they use more medication and make more doctor visits [5-6], they save less money, and up to 9% of caregivers need to quit their jobs [7]. Considering the setbacks suffered in the research on viable pharmacological treatments to counteract the progression of different types of dementia [8-11], the serious risks associated with using antipsychotic medications to treat the behavioural and psychological symptoms of dementia (BPSD) [12-14], and the high costs of overprescribing anti-dementia drugs [15], the development of effective non-pharmacological interventions to integrate or substitute the use of medications is of particular importance to increase both the effectiveness and the economic sustainability of dementia care.

Given this background, resource allocation could be enhanced by shifting from a standard approach—focused on containing the impact of distressing symptoms on patients through medications—to a more comprehensive approach based on the notion of person-centred care. This new approach would also follow the patient throughout the whole course of the disease by providing personalised care as well as support to patients and families. Defined as *supportive care* [16] and representing "a full mixture of biomedical dementia care, with good quality, person-centred, psychosocial, and spiritual care," this approach must be extended throughout the course of the illness to guarantee the overall wellbeing of PwDs and their caregivers [17]. The term supportive care (SC) refers to a wide array of non-pharmacological interventions that encompass a broad and growing range of services that are delivered either to the patient, the caregiver, or the patient-caregiver dyad [18-19]. This approach has been previously experimented in cancer care for addressing the clinical and psychosocial needs of patients in order to provide optimal quality of life [20] and in end-of-life care for non-cancer patients [21]. One of its key aspects is the decreasing reliance on medications that do not offer a sufficient benefit relative to the risks they pose in favour of novel non-pharmaceutical interventions [22]. In the case of PwDs,

SC is characterised by the continuous assistance of patients and their relatives from diagnosis until death, a holistic and interdisciplinary approach to care, and a high level of flexibility in choosing the right care practices for each case [23]. It is therefore evident that this definition of SC should not be confused with the one sometimes adopted in the cost-effectiveness literature, where the term "best supportive care" is used to denote care as usual or non-intervention.

A key feature of SC in all its stages is the central role of both formal and informal caregivers: the former are meant to have in-depth knowledge and competencies to deal with dementia patients, while the latter need to be recognised as indispensable players in dementia care, and both need to form and maintain collaborative relationships to guarantee high-quality care to patients [24]. In particular, support to caregivers could be considered a win-win solution, as it is beneficial for carers, patients, and the sustainability of healthcare systems [25]. On the opposite side of the spectrum, the unregulated use—and sometimes abuse—of antipsychotic drugs is a no-win situation, as it is detrimental for the health of the patient, and it puts a strain on the budget of healthcare systems [22]. In particular, regarding the management of behavioural problems in Alzheimer's disease, Gauthier and colleagues suggest that non-pharmacological interventions (including psychosocial/psychological counselling as well as interpersonal and environmental management) should be attempted first, followed by the least harmful medication for the shortest time possible [26].

Over the last few years, the evidence base on the effectiveness of non-pharmacological interventions (in terms of cognitive functioning and the reduction of behavioural symptoms of PwDs) has grown considerably [18-19]. Conversely, evidence on the value for money of non-pharmacological and SCIs for PwDs and their caregivers is still scant, despite the growing need for health care systems to base resource allocation decisions on cost-effective intervention strategies.

In this paper, we provide a systematic review of the main evidence on the cost-effectiveness of non-pharmacological and supportive practices for dementia, which may represent a safer, effective, and possibly more efficient alternative to traditional interventions based on pharmacological control of the behavioural symptoms associated with dementia, with the bonus that both patient and caregiver can benefit from some of these interventions at the same time.

Previous systematic reviews have highlighted the scarcity of economic evidence on non-pharmacological interventions for PwDs [27-28] and their caregivers [29]. These reviews were focused mainly on interventions

for community-dwelling persons with mild to moderate dementia [30] and home support interventions [31]. In contrast, we try to offer an all-encompassing review of cost-effectiveness studies on non-pharmacological and psychosocial interventions that target PwDs, their caregivers (either formal or informal), or the patient-caregiver dyad, and which are located at different stages of the care pathway for dementia and in different settings. Moreover, our systematic review is more complete since it provides evidence on the cost-effectiveness of indirect interventions such as organisational changes and innovations in the delivery of care and support.

Alternative strategies in dementia care: the increasing role of supportive care interventions

The global costs of Alzheimer's disease, the most prevalent subtype of dementia, and other dementias were estimated at US$818 billion in 2015 (equivalent to 1.09% of the global gross domestic product). These costs overcame the threshold of US$1 trillion in 2018 and are projected to double by 2030 [1,32]. Direct medical care represents the smallest share of the worldwide costs (19.5%), while direct social care and informal care contribute to the total in similar proportions (40.1% and 40.4%, respectively). The costs are concentrated in countries with higher income levels, and there is an imbalance between the global distribution of prevalence: 58% of people with dementia live in low- and middle-income countries, while 86% of costs occur in high-income countries. This is explained by the lower per-person costs in low- and middle-income countries due to lower wage costs and a high proportion of care provided by informal unpaid carers. However, current estimates often overlook important components of the socio-economic burden of dementia, such as the cost of health care for caregivers (due to anxiety or depression as a result of caring for someone with dementia), reduced quality of life (QoL) for PwDs and their carers, and the hidden costs that stack up before a diagnosis of dementia is even made [33].

Dementia-related costs are driven by the increase in life expectancy and the increased prevalence of cognitive impairment. Since demographic change is reducing the economically active part of the population, this poses a serious threat to the sustainability of social and health care systems [32-35]. There is consequently an urgent

need to find cost-effective bundles of medical and social care interventions that meet the needs of PwDs and their caregivers.

Regarding current drug therapies for dementia, cholinesterase inhibitors provide small but clinically important symptomatic benefits on cognition and function for persons with Alzheimer's disease, Lewy body dementia, and Parkinson's disease dementia. Moreover, in persons with severe Alzheimer's disease and for people with moderate disease who cannot tolerate cholinesterase inhibitors, memantine has been shown to have a small effect on cognition. However, these medications do not significantly change the course of illness and may provoke side effects [2, 8, 9], while only a limited number of ongoing clinical trials are currently investigating the viability of drugs directed at diverse therapeutic targets (e.g., amyloid and tau pathological processes, inflammatory pathways, or glial cells) [9-11].

Available medications for treating PwDs have been shown to enhance the QoL for both the patient and caregiver when prescribed at the appropriate time during illness. In particular, cholinesterase inhibitors—not only in the mild to moderate stage but also when symptoms become more severe—are more cost-effective than placebo and probably also cost-saving (by delaying the onset of institutionalisation), while the evidence in support of the cost-effectiveness of combination therapy (a cholinesterase inhibitor plus memantine) is less clear [36-37]. Conversely, there are potentially serious risks associated with using antipsychotic medications to treat BPSD [12-14], and there is no economic case for their use; moreover, there is no clinical or economic case for using antidepressant drugs to treat people with Alzheimer's disease who have comorbid depression [37].

A problem with antipsychotic medications is that these drugs do not offer a sufficient benefit relative to the risks they pose [25]; in fact, high prescription rates of antipsychotic drugs are putting dementia patients at risk of death and other adverse events. For example, a UK study shows that patients who received an antipsychotic treatment for 12 months were significantly more likely to have died by the 24-month and 36-month follow-up periods compared to patients who had received a placebo [38], while other studies have found a link between the use of antipsychotic drugs in dementia patients and an increase in motor symptoms and the risk of stroke and chest infections. Moreover, in many instances, antipsychotics are unnecessarily used to treat patients with only mild symptoms that could be addressed in other ways [22]. Despite this evidence in conjunction with the

warnings and best practice guidelines that have followed [12-14], the use of antipsychotic drugs is still widespread.

Non-pharmacological/SC interventions have emerged in the past few years as alternative and safer strategies to address BPSD and to enhance the QoL of both PwDs and their caregivers [16-17]. These interventions encompass a broad and growing range of services that are delivered either to the patient, the caregiver, or the patient-caregiver dyad; they stem from a variety of disciplines and their aim is to positively influence cognition, mood, agitation, activities of daily living (ADLs), and other symptoms of dementia [18-19].

A unified classification of SCIs for dementia does not exist yet, but several classifications can be found in the literature. For instance, Cammisuli et al. distinguish between holistic techniques, brief psychotherapy, cognitive methods, and alternative strategies [39]; in contrast, D'Onofrio et al. distinguish between cognitive and emotion-oriented interventions, sensory and multi-sensory stimulation interventions, and other interventions [40]. The World Alzheimer Report 2011 [41] and Nickel et al. [30] classified non-pharmacological interventions into four categories: physical exercise, interventions to support and enhance cognitive abilities, psychological and behavioural therapies, and occupational therapy.

In this paper, we distinguish between five categories of non-pharmacological/SC strategies: 1) cognitive therapies; 2) physical activity interventions; 3) indirect strategies; 4) interventions primarily targeted towards caregivers; 5) and multicomponent interventions.

We classify as cognitive therapies all those methods that stimulate a patient's cognition and may also control BPSD in several ways, including the following:

- Cognitive stimulation therapy (CST) is an evidence-based rehabilitation technique to enhance residual cognitive abilities and functional skills and preserve implicit memory [42]; patients are involved in activities such as word association games, quizzes, number games, physical games, and creative activities [43];
- Occupational therapy has the primary focus of preserving patients' independence by improving their ability to perform ADLs and adapt to their living environment; it can also be administered in a home setting by trained caregivers [44];

- Reality orientation therapy (ROT), one of the most popular psychosocial interventions to manage dementia, has the main goal of spatially and temporally reorienting patients, but it also helps the patient to maintain social interaction [45-46];
- Reminiscence therapy encourages patients to recall and talk about past experiences and events in their lives, either in individual or group sessions, and with the aid of props like photographs and videos [47];
- Learning therapy is a combined form of cognitive training and stimulation (adopted especially in Japan and the U.S.), where instructors help patients to perform simple calculations or reading tasks with face-to face verbal communication [48];
- Art or music therapy entails the therapeutic use of art or music to provide a dementia patient with meaningful stimulation and improve her/his participation and level of self-esteem [49]; one example is the global music approach to dementia (GMA-D), a patient-tailored music intervention [50];
- Intergenerational activities are those in which children and people with dementia can interact with each other to improve the patients' social interaction and sense of purpose [51];
- Doll and plushie therapy is usually used on patients with advanced dementia and involves the patient in behaviours such as holding, cuddling, feeding and dressing dolls or plushies [52];
- Pet therapy consists of the interaction of patients with animals, including activities such as petting, feeding, and playing with dogs and other animals [53].

Physical activity interventions include walking groups, aerobic exercise, and resistance training. These interventions can produce health benefits for patients, such as decreasing the number of falls and improving sleep and mood [54-56].

Indirect strategies include organisational and environmental changes, together with innovations in the delivery of care and support:

- Dementia care management programmes are defined as interventions delivered in the community aiming to coordinate the treatment and care for PwDs with respect to their needs and the recommendations of evidence-based guidelines [57-59];

- Dementia Care Mapping (DCM) is an observational tool that assists in the delivery of better formal care to PwDs, allowing for the adoption of a person-centred care approach to improve the quality of care of dementia patients [60];
- The Managing Agitation and Raising Quality of Life (MARQUE) intervention, a manual-based intervention targeted at the staff of care homes, is designed to train them in the implementation of procedures to reduce agitation in dementia patients [61];
- Memory clinics are facilities that provide guidance, prescriptions, rehabilitation, and various non-pharmacological interventions to dementia patients [62];
- Group living interventions allow patients with a similar level of cognitive impairment to cohabite in a controlled environment [63].

Interventions primarily aimed at caregivers include the following:

- Respite care refers to any kind of arrangement that provides short-term relief to primary caregivers by providing the patient with an alternative source of care and supervision; adult day centres and nurse visits are both forms of respite care [64-65];
- Programmes for caregivers include any intervention, usually of a psychosocial nature, that is primarily aimed at the primary caregivers; examples are support groups, family meetings, and coping strategies [66-70].

Lastly, we classify as multicomponent interventions the protocols that combine two or more different interventions among those described above. Examples of multicomponent interventions are the following:

- The WHELD (Wellbeing and Health for People with Dementia) intervention is implemented within a person-centred care framework; it includes physical exercise, approaches to reduce agitation, and psychosocial activities [71-72];
- Integrated approaches consist of personalised bundles of non-pharmacological interventions for the patient-caregiver dyad that are chosen after mapping their needs [73-74];
- Multicomponent support programmes are targeted at couples for whom one of the spouses suffers from dementia; they are support programmes that include group meetings for the caregivers, scheduled assessments by a geriatrician, and individualised services for the couple [75];

- Journeying through Dementia (JtD) is an intervention targeting the early stages of dementia that combines occupational therapy with self-management and peer support [76].

Materials and methods

Search strategy and criteria for inclusion

A systematic literature review was performed between February 2019 and March 2020 on the healthcare electronic databases MEDLINE (PubMed) and CDSR (Cochrane Database of Systematic Reviews). The search terms used to identify the articles to include in the review were as follows: (dementia OR alzheimer*) AND ('cost-effectiveness'/exp OR 'cost-analysis'/exp OR 'cost-utility'/exp) AND ('non-pharmacological'/exp OR psychosocial* OR 'drug-free'/exp). Additional details on the electronic search strategy can be found in the S1 File.

Study eligibility was based on the following criteria:

- Studies evaluating dementia interventions of any kind but the pharmacological one;
- Interventions aimed at either the patient or the caregiver (or the dyad patient-caregiver);
- The participants in the study had a diagnosis of dementia or were caregivers of a person with diagnosed dementia;
- Studies focusing on common and objective outcome measures for this area of research, to maximise comparability;
- Studies including an economic evaluation (a cost-effectiveness analysis and/or cost-utility analysis) or at least sufficient information on outcomes and costs to derive an incremental cost-effectiveness ratio (ICER) [77];
- Studies with an abstract in English.

The search strategy and the following review are both based on PRISMA recommendations [78]. The outcomes of the study selection process are described in the Results section.

Data collection and analysis

Data extraction was performed according to the guidelines of the Centre for Reviews and Dissemination for reviews of economic evaluations [79]; information was collected on the type of economic evaluation, study objective, study design, description of the intervention, comparators, measures of benefit and cost, and outcome and cost results.

The high heterogeneity in terms of interventions and outcome measures of the studies evaluated made it impossible to perform a meta-analysis, so we proceeded with a qualitative analysis.

Quality appraisal of included studies

The methodological quality of the studies included in the review was assessed using the Consensus on Health Economic Criteria (CHEC) checklist [80], which consists of 19 yes-or-no questions. To each study, we assigned a score from 0 to 19 based on the number of questions that the assessor answered with a "yes". Studies were classified as being high-quality if the score was equal to or higher than 17, medium-quality studies were those with a score between 14 and 16, and low-quality studies were those which scored 13 or lower. The score also reflects the information contained in additional analyses for those that actively pointed to other articles for additional information on the study design and/or protocol. The principal reviewer (AG) assessed the quality of all the articles, and the other four members of the research team (VR, OP, MR, and GB) checked for accuracy within their subsets. Any disagreement was resolved through discussion or consultation. The quality appraisal was undertaken to aid in interpreting the findings and determining the strength of the conclusions drawn; no study was excluded based on the results of the quality assessment.

Main outcomes of supportive care interventions

As mentioned above, the outcome indicators considered in the studies included in the review were highly heterogeneous even though it is possible to broadly distinguish between patient and caregiver outcome measures.

The main patient outcomes considered in the analysed studies were the following:

- Quality of life – the cognitive and functional decline brought about by dementia has a huge impact on the patient's QoL, and most studies include both generic health-related QoL (HRQoL) and dementia-related QoL as outcomes [81];
- Cognitive impairment – dementia impacts short- and long-term memory but also other cognitive functions such as language, abstract thinking, and judgement [82];
- Dementia severity – the gradual progression of the disease is measured with staging instruments that monitor the clinical and cognitive deterioration caused by dementia [83];
- Behavioural and psychological symptoms of dementia (BPSD) – these neuropsychiatric disturbances, such as apathy or hallucinations (or other non-cognitive symptoms), constitute a major component of dementia and have an impact on QoL [84];
- General health – this variable is gauged by looking at comorbidities, adverse events (i.e., untoward medical occurrences in a patient, including falls and fractures), nutritional status, etc. [85-86];
- Mental health – this variable can be measured by looking at an individual's depression levels, anxiety levels, schizophrenic or psychotic episodes, etc. [87];
- Agitation – as one of the most commonly observed neuropsychiatric symptoms in patients suffering from dementia, this condition is described as restless behaviour or improper physical and/or verbal action that can be a source of trouble for others [88];
- ADLs and IADLs – the number of (instrumental) activities of daily living an individual is able to carry out in an accepted way is a measure of functional capacity, which is an important indicator of health in the elderly [89];
- Prescription drug use – the use of antipsychotic medications to treat the BPSD;

- Service utilisation (and related costs) – the extent to which dementia patients use medical and/or social services and resources [90], including institutionalisation [91].

For each patient outcome, Table 1 reports the correlate measures considered in the selected studies.

Table 1. Patient outcomes and their measures in the reviewed studies.

Outcome	Measures
Health Related Quality of life (HRQoL)	• EuroQol (EQ-5D)<br>• Short Form-12 Health Survey (SF-12)<br>• Index of Well-Being (IWB)<br>• Rosser index
Dementia-Related Quality of life	• Dementia Quality of Life (DEMQOL)<br>• Quality of Life in Alzheimer's Disease (QoL-AD)
Cognitive impairment	• Mini-Mental State Examination (MMSE)<br>• Alzheimer's Disease Assessment Scale – Cognitive Subscale (ADAS-COG)<br>• Verbal fluency test (VF)<br>• Clock drawing test (CDT)<br>• Frontal Assessment Battery (FAB)<br>• Autobiographical Memory Interview (AMI)
Dementia severity	• Clinical Dementia Rating (CDR)<br>• Functional Assessment Staging of Alzheimer's Disease (FAST)
Behavioural and psychological symptoms	• Neuropsychiatric Inventory (NPI)<br>• Behavioural and Psychological Symptoms of Dementia (BPSD)<br>• Revised Memory and Behavior Problems Checklist (RMBPC)
General health	• Short Form-12 Health Survey (SF-12)<br>• General Health Questionnaire (GHQ)<br>• Charlson Comorbidity Index (CCI)<br>• Disability Assessment for Dementia (DAD)<br>• Falls and fractures<br>• Institutionalisation rates<br>• Certification of Needed Long-Term Care (CNLTC)<br>• COOP WONCA Functional Status Assessment Charts<br>• Mini Nutritional Assessment (MNA)
Mental health	• Hospital Anxiety and Depression Scale (HADS)<br>• Global Deterioration Scale (GDS)<br>• Cornell Scale for Depression in Dementia (CSDD)<br>• Rating of Anxiety In Dementia (RAID)<br>• MOS 20-Item Short Form Survey Instrument – Mental Health (MOS-20MH)
Agitation	• Cohen-Mansfield Agitation Inventory (CMAI)
Activities of daily living	• Alzheimer's Disease Cooperative Study ADL Scale (ADCS-ADL)<br>• Barthel Index (BI)<br>• Bristol Activities of Daily Living Scale (BADLS)<br>• Groningen Activities Restriction Scale (GARS)<br>• Lawton Brody scale (IADLs)<br>• Assessment of Motor and Process Skills (AMPS)

	• Interview for Deterioration in Daily Living Activities in Dementia (IDDD)<br>• Katz scale (ADLs)<br>• Multi-Dimensional Dementia Assessment Scale (MDDAS)
Use of prescription drugs	• Use of antipsychotics
Service utilisation	• Client Service Receipt Inventory (CSRI)<br>• Resource Utilization in Dementia (RUD)<br>• Institutionalization rates

The main caregiver outcomes considered in the reviewed studies were the following (see Table 2 for details on the different measures for each outcome):

- Quality of life – dementia severely impacts the QoL of caregivers because caring for someone who suffers from dementia is extremely burdensome and contributes to physical and psychiatric illnesses [92];
- Burnout and burden – caregiver burden is the perceived negative effect of caring for a family member [93], while caregiver burnout is more specifically a state of physical, emotional, and mental exhaustion [94];
- Sense of competence and mastery – competence is the extent to which a caregiver feels he or she can effectively do what is needed for a patient, whereas mastery is the extent to which a caregiver feels in control of the situation; both have been linked to positive outcomes for the caregiver [95-96];
- General health – caregivers are more likely to report poor health because they have less time to take care of themselves and face substantial stress (as indicated by the increased levels of cortisol) [97-98];
- Mental health – depression is very common among dementia caregivers, as are sleep disturbances, loneliness, and social isolation [99-100];
- Quality of interaction with the patient – low-quality interactions can undermine both the caregiver's QoL and quality of care [101]; the quality of the relationship that occurs between the caregiver and the patient has been found to be predictive of outcomes like the patient's institutionalisation and functional decline [102-103];
- Coping strategies – coping strategies employed by caregivers, such as avoidance or wishful thinking, are linked to physical and mental health outcomes [104];

- Time spent caregiving – caring for a PwD is not only a burdensome task, but it is also time consuming, as it prevents informal caregivers from having a regular work-life balance [105];
- Service utilisation – the additional medical and social service use by caregivers themselves helps us better understand the impact dementia has on societal costs [106];
- Absenteeism – formal and informal dementia caregivers are more likely to have higher absenteeism rates [107].

Table 2. Caregiver outcomes and their measures in the reviewed studies.

Outcome	Measures
Health Related Quality of Life (HRQoL)	• EuroQol (EQ-5D)<br>• Short Form-12 Health Survey (SF-12)<br>• World Health Organization Quality of Life Brief Version (WHOQoL-BREF)<br>• RAND 36-Item Health Survey (RAND-36)
Care Related Quality of Life	• Caregiver Quality of Life Instrument (CQLI)
Caregiver burden and burnout	• Maslach Burnout Inventory (MBI)<br>• Zarit Burden Interview (ZBI)
Sense of competence and mastery	• Sense of Competence in Dementia Care (SCID)<br>• Sense of Competence Questionnaire (SCQ)<br>• Pearlin Mastery Scale (PMS)
General health	• Short Form-12 Health Survey (SF-12)
Mental health	• Relative Stress Scale (RSS)<br>• Mini International Neuropsychiatric Interview (MINI)<br>• Centre for Epidemiologic Studies Depression Scale (CES-D)<br>• State Trait Anxiety Inventory (STAI)
Quality of relationship	• Quality of Interactions Schedule (QUIS)<br>• Quality of Carer and Patient Relationship scale (QCPR)
Coping strategies	• COPE inventory
Time spent caregiving	• Caregiving time spent doing things<br>• Caregiving time spent being on duty<br>• Resource Utilisation in Dementia questionnaire
Service utilisation	• Health Services Utilization Questionnaire (HSUQ)
Absenteeism	• Time away from work

Results

Study selection

The systematic search identified 3,218 publications. Duplicate citations were removed using Endnote X9, resulting in a total of 1,182 publications. After an initial screening of the titles and abstracts, 198 publications remained. After applying the eligibility criteria, 41 publications remained for full-text screening. A further 3 articles were added after screening the references of the reviewed articles. A final sample of 33 studies remained for inclusion in the review, including a study in the Dutch language [73]. The search strategy, based on PRISMA recommendations [78], is shown in the flow chart in Fig 1.

Fig 1. PRISMA flow chart of the study selection process.

(FIGURE 1 SHOULD BE INSERTED HERE)

Characteristics of the included studies

We reviewed 33 studies that analysed 29 interventions: nine cognitive stimulation and occupational programmes primarily targeted at PwDs; two physical activity interventions; eight indirect interventions (organisational and environmental changes); six interventions primarily targeted towards carers; and four structured multicomponent interventions. Several SC programmes considered outside the formal category of "multicomponent interventions" may however include more elements when based on structured protocols combining different components; for example, the community-based occupational therapy programme directed at community-dwelling patient-caregiver dyads analysed by Graff et al. [108] included treatment at home of PwDs but also education and behaviour management for informal caregivers. The most common component across the reviewed interventions was behaviour management for carers, which was addressed in 11 studies.

The interventions analysed in the studies were located at different stages of the care pathway for dementia: 12 studies focused on patients with dementia in its mild to moderate stages and/or their caregivers [59,62,67,74,108-115]; 17 interventions addressed the moderate-to-severe stages [61, 63-66, 68, 71-73, 116-123]; and four studies focused on PwDs at different stages [58, 75, 124-125]. Six studies considered SCIs in nursing homes and assisted living settings [61, 71-72, 119-121], and two studies analysed both residential and community settings [111, 124], while the rest of the health economic analyses concerned community-based interventions. Most studies analysed SCIs directed at patient-caregiver dyads, while seven studies focused on specific programmes supporting informal caregivers of community-dwelling PwDs [64, 66-68, 117, 122, 123]. Most studies (27 out of 33, and 23 out of 29 interventions) were conducted in European countries with comparable underlying health and social care systems (14 were based in the UK), while only six studies were developed in other OECD countries, including three in the United States, one in Japan, one in Australia, and one in Canada.

The studies were quite heterogeneous in terms of their design, the cost items included, and the choice of outcome measures. One study [63] used data from secondary sources in a Markov model to estimate the cost-utility of the intervention. Most studies (n = 26) used a randomised controlled trial (RCT) design, while five were non-randomised comparisons through prospective matched controlled trials [58, 63, 65, 119, 122]. Eighteen of the analysed studies adopted a narrow perspective when measuring costs, looking only at health and social care, while the other 15 studies considered a broader societal perspective, including the opportunity costs of caregivers' inputs and the impacts of caring on their own health and wellbeing.

In all the analysed studies, the SC intervention under investigation was explicitly compared—with regard to costs and outcome measures—with one or more alternatives (in most cases, the "usual care" alternative). Most studies, except for four [75, 115, 119, 121], reported the ICER of SCIs. The ICER is calculated according to the following formula:

$$ICER = \Delta C / \Delta E$$

where ΔC is the difference in mean costs between the intervention under evaluation and the comparator, and ΔE is the mean difference in outcome between the intervention under evaluation and the comparator. When the outcome is measured in terms of utility values to account for the patient's and/or carer's QoL (e.g., using the Quality Adjusted Life Years [QALYs] gained), the cost-effectiveness analysis takes the form of a cost-

utility analysis. Some studies [111, 114, 119, 124-125] calculated the net-benefits (NB) of supportive care interventions using a series of hypothetical values for the decision maker's willingness-to-pay (ω) for an additional unit of outcome (e.g., a one-point difference in the Neuropsychiatric Inventory [NPI] score):

$$NB = \omega * \Delta E - \Delta C$$

Ten studies were pure cost-effectiveness analyses [65, 68, 71, 108-109, 113, 116, 120, 122, 124], ten were cost-utility analyses [59, 61-64, 67, 73-74, 114, 123], ten developed both a cost-effectiveness and a cost-utility analysis [58, 66, 72, 110-112, 115, 117-118, 125], two were cost-consequence analyses [75, 121], and one was a cost-benefit analysis [119].

Quality assessment of the included studies

As indicated above, we also assessed the methodological quality of the studies included in the review using the CHEC checklist [80]. Based on the scores assigned, studies were classified as being high-, medium-, or low-quality. The quality level of the study is reported in the last columns of Tables 3, 4, and 5. Overall, only two studies [59,123] met all 19 criteria defined in the checklist. Applying the CHEC criteria described in the Materials and methods section, we found 11 high-quality studies, 15 medium-quality studies, and 7 low-quality ones. Details on the ratings of the studies can be found in Supporting information (S1, S2, and S3 Tables).

Evidence of cost-effectiveness of supportive care interventions from reviewed studies

In this section, we describe the results of the qualitative analysis of the studies considered in the systematic review by distinguishing between studies providing evidence of high cost-effectiveness of SCIs (Table 3), those showing evidence of moderate cost-effectiveness (Table 4), and those showing very little or no evidence of cost-effectiveness (Table 5).

Tables 3, 4, and 5 report the main characteristics of the analysed studies, including type of assessed intervention (e.g., cognitive, physical activity, indirect, or multicomponent); description of the intervention under

evaluation; country where the intervention was implemented; type of study, type of economic evaluation, and time horizon; sample size (i.e., number of PwDs and caregivers considered in the study as well as the size of intervention and control groups); patient outcome measures; caregiver outcome measures; mean ICER or other cost-effectiveness measures (e.g., the intervention's net benefit); and assessed quality of the study.

Interventions with evidence of high cost-effectiveness

Out of the 33 studies included in the systematic review, 10 provided evidence of the high cost-effectiveness of SCIs (see Table 3).

Table 3. Studies that find evidence of high cost-effectiveness of supportive care interventions.

**Study**	**Type of intervention**	**Intervention Description and Comparator**<br><br>**(Setting)**	**Country**	**Type of study and economic evaluation**<br><br>**(Time horizon)**	**Sample size**<br>No. PwDs/No. Caregivers<br>(Intervention Group/Control Group)	**Perspective**	**PwD Outcome measures**	**Caregiver Outcome measures**	**Incremental Cost-Effectiveness Ratio (ICER)**<br>**and other cost-effectiveness measures**	**Quality of the study**
Gitlin et al. 2010 [116]	Cognitive	Tailored Activity Program (TAP) for patients and caregivers (occupational therapy)<br>vs Wait-list<br><br>(Community-based: home)	USA	RCT<br>CEA<br><br>(4 months)	60 / 60<br><br>(30 / 30)	Societal	----	•Caregiving time spent "doing things"<br>•Caregiving time spent "being on duty"	$2.37 per hour saved/day ("doing things"),<br>$1.10 per hour saved/day ("being on duty")	Low
Graff et al. 2008 [108]	Cognitive	Community occupational therapy (including cognitive and behavioural interventions)<br>vs Usual Care<br>(Community-based: Memory clinics, Day clinics of a geriatrics department, home)	Netherlands	RCT<br>CEA<br><br>(6 weeks;<br>3 months)	135 /135<br><br>(68 /67)	Societal	•Daily functioning (AMPS; IDDD)	•Sense of competence (SCQ)	€1,748 saved compared with control (difference in mean total care costs per successful treatment)	High
Wimo et al. 1995 [63]	Indirect	Group living for dementia patients<br>vs Home living and Institutional living<br><br>(Group living)	Sweden	Markov model<br>CUA<br><br>(Expected life-length of 8 years)	108 / 0<br><br>(46 / 39;23)	Health and social care system	•Degree of dementia (GDS)<br>•QALYs gained (IWB scale)	----	Incremental cost per QALY gained < 0 (intervention less costly and more effective)	Medium
Melis et al. 2008 [109]	Indirect	Dutch Geriatric Intervention Programme (nurse visits)<br>vs Usual Care<br><br>(Community-based: home)	Netherlands	RCT<br>CEA<br><br>(6 months)	151 / 0<br><br>(85 / 66)	Health and social care system	•IADLs (GARS-3)<br>•Mental well-being (MOS-20MH)	----	€3,500 per successful treatment	Medium
Nichols et al. 2008 [68]	For Caregivers	Psychosocial intervention for caregivers (REACH II)<br>Vs Usual care<br><br>(Community-based: home)	USA	RCT<br>CEA<br><br>(6 months)	112 / 112<br><br>(55 / 57)	Societal	•Cognitive impairment (MMSE)<br>•Behavioural and psychological symptoms (RMBPC)<br>•ADLs (Katz scale)<br>•IADLs (Lawton Brody scale)<br>•Service utilisation	•Time spent caregiving<br>•Caregiver bother (RMBPC)<br>•Depression (CES-D)<br>•Service utilisation<br>•Social support	$4.96 per hour not spent in caregiving	Medium

Study	Type of intervention	Intervention Description and Comparator<br><br>(Setting)	Country	Type of study and economic evaluation<br><br>(Time horizon)	Sample size No. PwDs/No. Caregivers (Intervention Group/Control Group)	Perspective	PwD Outcome measures	Caregiver Outcome measures	Incremental Cost-Effectiveness Ratio (ICER) and other cost-effectiveness measures	Quality of the study
Knapp et al. 2013 [66]	For Caregivers	Individual therapy program for caregivers (START) vs Usual Care alone<br><br>(Community-based: mental health and neurological outpatient dementia services)	UK	RCT CEA+CUA<br><br>(8 months)	260 / 260<br><br>(173 / 87)	Health and social care system	•Behavioural and psychological symptoms (NPI)	•Depression and anxiety (HADS)<br>•Quality of life (EQ-5D)<br>•Caregiver burden (ZBI)<br>•Coping strategies (COPE)	£6,000 per QALY gained (caregiver), £118 per HADS point (caregiver)	High
Livingston et al. 2014 [117]	For Caregivers	Individual therapy program for caregivers (START) vs Usual Care alone<br><br>(Community-based: mental health and neurological outpatient dementia services)	UK	RCT CEA+CUA<br><br>(24 months)	209 / 209<br><br>(140 / 69)	Health and social care system	•Dementia severity (CDR)<br>•Quality of life (QoL-AD)<br>•Behavioural and psychological symptoms (NPI)<br>•Service utilisation (CSRI)	•Depression and anxiety (HADS)<br>•Quality of life (EQ-5D)<br>•Caregiver burden (ZBI)<br>•Coping strategies (COPE)<br>•Service utilisation (CSRI)	£244 per QoL-AD point (patient)<br>£12,400 per QALY gained (caregiver)<br>£179 per HADS point (caregiver)<br>[carer-only costs]	Medium
Livingston et al. 2019 [118]	For Caregivers	Individual therapy program for caregivers (START) vs Usual Care alone<br><br>(Community-based: mental health and neurological outpatient dementia services)	UK	RCT CEA<br><br>(6 years follow-up)	222 / 222<br><br>(150 / 72)	Health and social care system	•Behavioural and psychological symptoms (NPI)<br>•Service utilisation (CSRI)	•Depression and anxiety (HADS)<br>•Caregiver burden (ZBI)<br>•Service utilisation (CSRI)	Mean difference in HADS scores of -2.00 points with no significant difference in patient and caregiver costs (intervention is more effective than TAU and costs the same)	Medium
Ballard et al. 2018 [71]	Multicomponent	WHELD intervention (person-centred care, management of agitation, physical exercise and psychosocial approaches) vs Usual Care alone<br><br>(Nursing Home)	UK	RCT CEA<br><br>(9 months)	553 / 0<br><br>(257 /296)	Health and social care system	•Quality of life (DEMQOL Proxy)<br>•Dementia severity (CDR, FAST)<br>•Agitation (CMAI)<br>•Behavioural and psychological symptoms (NPI)<br>•Mood (CSSD)<br>• Antipsychotic drug use<br>•Quality of interaction (QUIS)<br>•Service utilisation (CSRI)	----	Incremental cost per unit of effectiveness < 0 (intervention less costly and more effective)	Medium
Romeo et al. 2019 [72]	Multicomponent	WHELD intervention (person-centred care, management of agitation, physical exercise and psychosocial approaches) vs Usual Care alone<br><br>(Nursing Home)	UK	RCT CEA+CUA<br><br>(9 months)	549 / 0<br><br>(267 / 282)	Health and social care system	•Agitation (CMAI)<br>•Quality of life (DEMQOL Proxy)<br>•Service utilisation (CSRI)	----	-£137,978 per QALY gained<br>-£348 per CMAI point (intervention less costly and more effective)	Medium

Legend

ADLs: Activities of Daily living; AMPS: assessment of motor and process skills-process scale; CDR: Clinical Dementia Rating; CES-D: Center for Epidemiologic Studies Depression Scale; CMAI = Cohen- Mansfield Agitation Inventory; COPE: self-completed measure of carer coping strategies; CSRI: Client Service Receipt Inventory; CSSD: Cornell Scale for Depression in Dementia; CUA: Cost-utility analysis; DEMQOL Proxy: Dementia Quality of Life score reported by a carer; EQ-5D: EuroQol-5 Dimensions; GARS-3: Groningen Activity Restriction Scale-3; GDS: Global Deterioration Scale; HADS: Hospital Anxiety and Depression Scale; IADLs: Instrumental Activities of Daily Living; IDDD: interview of deterioration in daily activities in dementia-performance scale, measures need for assistance; IWB: Index of well-being; MMSE: Mini-Mental State Examination; MOS-20MH: mental health subscale of the Medical Outcomes Study Short Form; NPI: Neuropsychiatric Inventory; QALYs: Quality Adjusted Life Years; QoL-AD: Quality of Life-Alzheimer's Disease scale; QUIS: Quality of Interactions Scale; RCT: Randomised controlled trial; RMBPC: Revised Memory and Behavior Problem Checklist; SCQ: Sense of competence questionnaire; ZBI: Zarit Burden Interview (self-reported questionnaire used to assess carer burden).

Cognitive stimulation

Gitlin et al. [116] analysed an occupational therapy intervention referred to as the "Tailored Activity Program" (TAP), which was directed at community-dwelling patient-caregiver dyads. The effectiveness of TAP was measured in terms of the additional unit of benefit measured by caregiver hours per day of "doing things" and "being on duty" (i.e., TAP was able to reduce carer time inputs); accordingly, the ICER was expressed as the cost to bring about one additional unit of benefit. The intervention was found to be cost-effective: the ICER was $2.37 per hour saved every day by the caregiver in terms of "doing things", and $1.10 per hour saved every day in terms of "being on duty". However, due to the absence of primary outcomes (no measures of patient and carer health and QoL were considered) and other drawbacks, the quality of evidence of cost-effectiveness according to the CHEC was rather low (see S1 Table in Supporting information).

Graff et al. [108] studied another form of community-based occupational therapy that included both behavioural and cognitive interventions; even in this case, the programme was aimed at patient-caregiver dyads. The intervention consisted of 10 sessions of occupational therapy administered over five weeks, including cognitive and behavioural interventions, to train patients in the use of aids to compensate for cognitive decline and caregivers in coping behaviours and supervision. The authors found average savings of approximately €1,748 per couple who had been successfully treated with the considered occupational therapy compared to usual care; the probability of occupational therapy being the dominant intervention (i.e., more effective and less costly) was estimated to be 94%. They concluded that the community occupational therapy intervention for PwDs and their caregivers was cost-effective, especially in terms of informal caregiving.

Indirect interventions

A Swedish prospective study [63] on group living for dementia patients with a Global Deterioration Scale (GDS) score between four and seven compared the cost-effectiveness of this type of intervention with that of living at home or in nursing homes. Group living is considered an intermediate level of care between home and institutionalisation. The authors applied a cost-utility analysis using a Markov model of an expected life-length of eight years. They considered a sample of 108 PwDs, for which 46 patients in group living were compared to 39 patients living at home and 23 institutionalised patients. They found the intervention to be

highly cost-effective and dominant (the ICER, measured in terms of cost per QALY, was negative) compared to both institutionalisation and living at home.

Melis et al. [109] evaluated the cost-effectiveness of the Dutch Geriatric Intervention Programme, consisting of regular nurse visits for community-dwelling frail older people, including PwDs. The difference in the treatment effect was calculated as the difference in the proportions of successfully treated patients (prevention of functional decline together with improved wellbeing). The ICER was expressed as the total incremental cost per successful treatment, and it was roughly €3,500 (-21,458 to 45,362). The intervention had a 95% probability of being cost-effective compared with usual care at a willingness-to-pay (WTP) threshold of €34,000.

Interventions for carers

Nichols et al. [68] performed a cost-effectiveness analysis on a psychosocial intervention (REACH II) for caregivers of community-dwelling dementia patients, consisting of individual sessions and telephone-administered support group sessions. The intervention included components that targeted five problem areas linked to caregiver risk and QoL: caregiver burden, emotional wellbeing, self-care and healthy behaviours, social support, and care-recipient problem behaviours. In this case, the ICER represented the cost of an additional hour of non-caregiving time that could be "purchased" by the intervention. There was no significant difference in formal healthcare use between the control and intervention dyads, while there was a significant reduction of hours of provided care for the caregivers in the intervention group compared to those in the control group. Nichols et al. found that the six-month intervention was cost-effective if one was willing to spend $4.96 per day for one extra hour of non-caregiving time for each caregiver; moreover, the intervention could be thought of as being financially positive because it resulted in $10.56 of time gained versus $4.96 of intervention cost per hour per day per caregiver.

Three studies [66, 117-118] analysed the cost-effectiveness of the "Strategies for Relatives" (START) intervention, an individual psychosocial therapy programme aimed at informal caregivers to help them cope with the illness faced by their non-institutionalised relative. This particular approach consists of an eight-session, manual-based coping intervention delivered by supervised psychology graduates to family carers of PwDs in addition to usual treatment. In one of these studies, Knapp et al. [66] examined the short-term (eight

months) cost-effectiveness of START, and they found that the START mean cost per QALY gained was £6,000, and the intervention had a greater than 99% chance of being cost-effective compared with usual treatment alone at a WTP threshold of £30,000 per QALY gained, which is within the threshold range of £20,000–30,000 per QALY currently used by the British National Institute for Health and Care Excellence (NICE) [126]. Moreover, START showed a high probability of cost-effectiveness on the HADS-T (Hospital Anxiety and Depression Scale) measure.

In another study, Livingston et al. [117] analysed the 24-month impact on patient and caregiver outcomes (with a follow-up rate of 80%) and found that START was cost-effective for both, with a 67% probability of cost-effectiveness at the £20,000 per QALY willingness-to-pay threshold and 70% at the £30,000 threshold. A 2019 follow-up by Livingston et al. [118] found that, after six years, the intervention remained clinically effective with no significant difference between the treated and the control group in terms of costs and time to care home admission or death; however, carers in the control group were five times more likely to have clinically significant depression.

Multicomponent interventions

A widely studied multicomponent strategy is the UK-based "Improving Wellbeing and Health for People with Dementia" (WHELD) intervention, which is targeted towards patients in nursing homes. This approach consists of a protocol to manage agitation coupled with physical exercise and psychosocial activities, all within a person-centred care framework. The protocol is focused on training care staff and promoting tailored person-centred activities and social interactions; it also involves the development of a system for triggering the appropriate review of antipsychotic medications by the prescribing physician.

A preliminary study by Ballard et al. [71] found evidence of the potential cost-effectiveness of WHELD. In particular, the intervention produced significant (albeit small) benefits in terms of patients' QoL measured with a Dementia Quality of Life (DEMQOL) proxy (i.e., the DEMQOL score reported by the carer), agitation, and overall neuropsychiatric symptoms, especially for people with moderately severe dementia; antipsychotic drug use was at a low stable level in both the treatment and control groups. Taking into account the cost of the intervention and the total health and social care costs, the authors found that the WHELD intervention reduced costs compared to usual care; therefore, the benefits achieved were associated with cost savings.

In another study, Romeo et al. [72] evaluated WHELD cost-effectiveness and cost-utility and found that the intervention was cost-effective compared to usual care alone across a wide range of WTP on the part of a decision maker for a unit improvement in outcome (the considered outcome measures were the Cohen-Mansfield Agitation Inventory [CMAI] and dementia-related QoL according to the DEMQOL-Proxy). The cost-effectiveness was mostly attributed to the lower health and social care costs faced by the intervention group compared to the control group. The authors also found that these results were mainly relevant to residents with clinically significant agitation in dementia.

Interventions with evidence of moderate cost-effectiveness

Evidence of the moderate cost-effectiveness of several SCIs was found in 14 of the analysed studies (see Table 4).

Table 4. **Studies that find evidence of moderate cost-effectiveness of supportive care interventions.**

Study	Type of intervention	Intervention Description and Comparator<br><br>(Setting)	Country	Type of study and economic evaluation<br><br>(Time horizon)	Sample size No. PwDs /No. Caregivers (Intervention Group/Control Group)	Perspective	PwD Outcome measures	Caregiver Outcome measures	Incremental Cost-Effectiveness Ratio (ICER) and other cost-effectiveness measures	Quality of the study
Clare et al. 2019 [110]	Cognitive	Cognitive rehabilitation (GREAT trial) vs Usual Care<br><br>(Community-based: home)	UK	RCT<br>CEA + CUA<br><br>(3 months<br>6 months)	427 / 427<br><br>(209 / 218)	•Health and social care system<br><br>•Societal	•Self-reported goal attainment (BGSI)<br>•Quality of life (DEMQOL)<br>•Depression and anxiety (HADS)<br>•Self-efficacy (GSES)<br>•Cognitive impairment measures<br>•Service utilisation (CSRI)	•Relatives' Stress Scale (RSS)<br>•Health status assessment<br>•Quality of life (EQ-5D, WHOQoL-BREF)	£1,296 per BGSI point<br>£1,110,000 per QALY gained (patient), £632,000 per QALY gained (caregiver)<br>(health and social care perspective)	High
Sado et al. 2020 [119]	Cognitive	Learning Therapy vs Usual Care<br><br>(Nursing Home)	Japan	Prospective study<br>CBA<br><br>(12 months)	57 / 0<br><br>(30 / 27)	Health and social care system	•Level of care needed (CT-CNLTC)<br>•Quality of life (EQ-5D)<br>•Cognitive impairment (MMSE, FAB)<br>•PMS/IADL	----	$1,605 as net monetary benefit	Medium
Mervin et al. 2018 [120]	Cognitive	Plushie robot (PARO) or normal plushie<br>vs Usual Care<br><br>(Nursing Home or other residential facility)	Australia	RCT<br>CEA<br><br>(10 weeks)	415 / 0<br><br>(138; 140 / 137)	Health and social care system	•Agitation (CMAI-SF)<br>•Medications	----	AU$13.01 per CMAI-SF point averted (PARO), AU$12.85 per CMAI-SF point averted (plushie)	Low
D'Amico et al. 2015 [111]	Cognitive	Maintenance cognitive stimulation therapy<br>vs Usual Care alone<br><br>(Different Settings: Nursing Home and Community Centre)	UK	RCT<br>CEA + CUA<br><br>(24 weeks)	199 / 0<br><br>(106 / 93)	•Health and social care system<br>•Societal	•Cognitive impairment (ADAS-Cog, MMSE)<br>•Quality of life (QoL-AD, DEMQOL, EQ-5D)<br>•Behavioural and psychological symptoms (NPI)<br>•ADLs (ADCS-ADL)<br>•Service utilisation (CSRI)	----	£26,835 per QALY, £266 per QoL-AD point, £558 per MMSE point	Medium
Orgeta et al. 2015 [112]	Cognitive	Carer-led individual cognitive stimulation therapy<br>vs Usual Care<br><br>(Community-based: Memory clinics and community mental health teams for older people)	UK	RCT<br>CEA+CUA<br><br>(13 weeks;<br>26 weeks)	273 / 273<br><br>(134 / 139)	•Health and social care system<br>•Societal	•Cognitive impairment (ADAS-Cog, MMSE)<br>•Quality of life (QoL-AD, DEMQOL-Proxy)<br>•Behavioural and psychological symptoms (NPI)<br>•BADLS<br>• Depression and anxiety (GDS),<br>• Relationship (QCPR)<br>•Service utilisation (CSRI)	•Mental and physical health (SF-12)<br>• Depression and anxiety (HADS)<br>Distress (NPI)<br>• Quality of life (EQ-5D)<br>• Carer resilience (RS-14)<br>• Relationship (QCPR)<br>•Service utilisation (CSRI)	£3,100 per QALY gained (caregivers)	High

Study	Type of intervention	Intervention Description and Comparator<br><br>(Setting)	Country	Type of study and economic evaluation<br><br>(Time horizon)	**Sample size** No. PwDs /No. Caregivers (Intervention Group/Control Group)	Perspective	PwD Outcome measures	Caregiver Outcome measures	Incremental Cost-Effectiveness Ratio (ICER) and other cost-effectiveness measures	Quality of the study
Knapp et al. 2006 [124]	Cognitive	Cognitive stimulation therapy vs Usual Care<br><br>(Different Settings: Nursing Home, Community and Day Centre)	UK	RCT<br>CEA<br><br>(8 weeks)	161 / 0<br><br>(91 / 70)	•Health and social care system	•Cognitive impairment (MMSE)<br>•Quality of life (QoL-AD)<br>•Service utilisation (CSRI)	----	£75.32 per MMSE point,<br>£22.82 per QoL-AD point	Medium
D'Amico et al. 2016 [125]	Physical activity	Physical exercise regimen (walking) for patient-caregiver dyads vs Usual Care<br><br>(Community-based: home)	UK	RCT<br>CEA+CUA<br><br>(12 weeks)	52 / 52<br><br>(30 / 22)	•Health and social care system<br>•Societal	•Behavioural and psychological symptoms (NPI)<br>•General health (GHQ)<br>•Quality of life (DEMQOL Proxy)<br>•Service utilisation (CSRI)	•Caregiver burden (ZBI)	£421 per NPI point, £1,055 per ZBI point, £286,440 per QALY gained (societal perspective)	Medium
MacNeil Vroomen et al. 2016 [58]	Indirect	Case management (Intensive Case Management Model or Linkage Model) vs Usual Care<br><br>(Community-based: home)	Netherlands	Prospective study<br>CEA+CUA<br><br>(24 months)	521 / 521<br><br>(234; 214 / 73)	•Societal	•Behavioural and psychological symptoms (NPI)<br>•Quality of life (EQ-5D)	•Mental health (GHQ)<br>•Quality of life (EQ-5D)	€9,581.433 per QALY (ICMM vs control),<br>€2,236,139 per QALY (LM vs control)<br>(combined QALY of patient and caregiver)	Medium
Michalowsky et al. 2019 [59]	Indirect	Dementia Care Management (Delphi-MV trial) vs Usual Care<br><br>(Community-based: home)	Germany	RCT<br>CUA<br><br>(24 months)	444 / 0<br><br>(315 / 129)	•Health and social care system	• Health related Quality of life (SF-12)<br>•Cognitive impairment (MMSE)<br>•Depression and anxiety (GDS)<br>•B-ADL<br>•Comorbidity (CCI)<br>•Service utilisation (CSRI)<br>•Time to institutionalisation	----	Incremental cost per QALY < 0<br>€26,851 per QALY (PwD living with a caregiver)	High
Rädke et al. 2020 [115]	Indirect	Dementia Care Management (Delphi-MV trial) vs Usual Care<br><br>(Community-based: home)	Germany	RCT<br>CUA<br><br>(24 months)	444 / 0<br><br>(315 / 129)	•Health and social care system	• Health related Quality of life (SF-12)<br>•Cognitive impairment (MMSE)<br>•Depression and anxiety (GDS)<br>•B-ADL<br>•Comorbidity (CCI)<br>•Service utilisation (CSRI)	----	Probability of cost-effectiveness at €40,000 per QALY is higher for females, patients living alone, patients with moderate dementia, patients with high comorbidities	High

Study	Type of intervention	Intervention Description and Comparator<br><br>(Setting)	Country	Type of study and economic evaluation<br><br>(Time horizon)	Sample size No. PwDs /No. Caregivers (Intervention Group/Control Group)	Perspective	PwD Outcome measures	Caregiver Outcome measures	Incremental Cost-Effectiveness Ratio (ICER) and other cost-effectiveness measures	Quality of the study
Wimo et al. 1994 [65]	Indirect	Adult Day Care vs Wait-list<br><br>(Community-based: Day Care)	Sweden	Prospective study<br>CEA<br><br>(12 months)	100 /0<br><br>(55 / 45)	•Health and social care system	•Quality of life (IWB, Rosser index)<br>•Cognitive impairment (MMSE)<br>•ADLs and Behaviour (MDDAS)	----	Incremental cost per unit of effectiveness < 0 for patients with severe psycho-social stress situation (intervention less costly and more effective)	Medium
Van de Ven et al. 2014 [121]	Indirect	Dementia Care Mapping<br>Vs Usual care<br><br>(Nursing Home)	Netherlands	RCT<br>CCA<br><br>(18 months)	318 / 319<br>(PwD: 154/164)<br>(Staff: 141/178)	•Health and social care system	•Service utilisation<br>• Antipsychotic drug use<br>•Falls and fractures	•Absenteeism	N.A.	Low
Gaugler et al. 2003 [122]	For Caregivers	Adult day care service to support caregivers<br>vs Usual Care<br><br>(Community-based: Day Care)	USA	Prospective study<br>CEA<br><br>(3 months; 1 year)	0 /201<br><br>(80/121)	Societal	•Behaviour Problem Scale<br>•ADL	•Stress (ROS)<br>•Depression (CES-D)	$4.51/day per unit of ROS score<br>$2.20/day per unit of CES-D score (over 1-year period)	Low
Wolfs et al. 2011 [73]	Multicomponent	Integrated approach (map of the patient and caregiver needs to develop a personalised treatment course)<br>vs Usual Care<br><br>(Community-based: Diagnostic research centre for psycho-geriatrics)	Netherlands	RCT<br>CUA<br><br>(1 year)	219/ 0<br><br>(131 /88)	Societal	•Quality of life (EQ-5D)<br>•Cognitive impairment (MMSE)<br>•Behavioural and psychological symptoms (NPI)<br>•IADLs (Lawton Brody scale)<br>•Deepression (CSDD)<br>•Service utilisation	----	€1,267 per QALY gained	Low

Legend

ADAS-Cog: Alzheimer's Disease Assessment Scale-Cognition subscale; ADCS-ADL: Alzheimer's Disease Cooperative Study-Activities of Daily Living Inventory; B-ADL: Bayer-Activities of Daily Living Scale; BADLS: Bristol Activities of Daily Living Scale; CCA: Cost consequence analysis; CCI: Charlson Comorbidity Index; CEA: Cost-effectiveness analysis; CES-D: Center for Epidemiologic Studies Depression Scale; CMAI-SF = Cohen-Mansfield Agitation Inventory-Short Form; CSDD: Cornell Scale for Depression in Dementia; CSRI: Client Service Receipt Inventory; CUA: Cost-utility analysis; DEMQOL: Dementia Quality of Life score; DEMQOL Proxy: Dementia Quality of Life score reported by a carer; EQ-5D: EuroQol-5 Dimensions; FAB: Frontal Assessment Battery; GDS: Geriatric Depression Scale; GHQ: General Health Questionnaire; GSES: Generalized Self-Efficacy Scale; HADS: Hospital Anxiety and Depression Scale; IWB: Index of well-being; MDDAS: Multi-Dimensional Dementia Assessment Scale; MMSE: Mini-Mental State Examination; NPI: Neuropsychiatric Inventory; PMS/IADL: Physical Self-Maintenance Scale/Instrumental Activity of Daily Living; QCPR: Quality of Caregiver–Patient Relationship; QoL-AD: Quality of Life-Alzheimer's Disease scale; RCT: Randomised controlled trial; ROS: Role Overload Scale; RS-14: Resilience Scale-14 items; RSS: Relatives' Stress Scale; SF-12: Short Form questionnaire-12 items; ZBI: Zarit Burden Interview (self-reported questionnaire used to assess carer burden); WHOQoL-BREF: World Health Organization's Quality of Life Instrument – brief version.

Cognitive stimulation

Clare et al. [110] developed a single-blind multicentre RCT (Goal-Oriented Cognitive Rehabilitation in Early-Stage Alzheimer's and Related Dementias Trial [GREAT]) in order to provide evidence of the clinical and cost-effectiveness of a cognitive rehabilitation programme for people with mild to moderate dementia and their caregivers. The individualised intervention consisted of 10 therapy sessions administered over three months, followed by four maintenance sessions over six months, delivered in participants' homes under the direction and assistance of a cognitive rehabilitation therapist. The therapists (nine occupational therapists and one nurse) worked with the patient and the carer to identify realistic and relevant goals, plan how to tackle them, and support people in achieving them. The primary outcome was the participant rating of goal attainment, and goals were elicited using the Bangor Goal-Setting Interview (BGSI), with goal attainment assessed using a validated rating scale on which a two-point improvement was considered to be clinically significant. For the intervention group, participant attainment ratings improved at the three-month follow-up by 2.57 points on average, and this improvement was maintained at nine months; average ratings in the TAU group showed a negligible improvement of less than one point at three months. Patient's and carer's QoLs (measured with several instruments) were considered a secondary outcome together with other measures; however, there were no significant changes in any secondary outcome measures following the intervention. Clare et al. also performed both a cost-effectiveness and a cost-utility analysis of the cognitive rehabilitation programme conducted, first, from a health and social care perspective and, second, from a societal perspective. The cost of an increase of 1.32 points in the BGSI attainment rating was £1,296 from the health and social care perspective and -£9 from the societal perspective; the intervention could be considered cost-effective in terms of achieving an improvement in participant-rated goal attainment, with the WTP being £2,500 and above under both the societal and healthcare system perspectives. However, in both studies, there was no evidence for cost-effectiveness either in terms of QALY gains for PwDs (measured with DEMQOL) or in terms of QALY gains for carers (measured with the EuroQol - 5 Dimensions [EQ-5D] three-level version).

Sado et al. [119] performed an initial cost-benefit analysis of learning therapy for nursing home patients, which is an intervention that combines different cognitive activities (e.g., simple math calculations, reading tasks, and conversations) undertaken under the guidance and acknowledgement of an instructor. The primary outcomes were safety, validity of eligibility, retention rate, and effect on the functions of daily living

represented by Criterion Time for Certification of Needed Long-Term-Care (CT for CNLTC) at 12 months. The authors offered preliminary evidence that the intervention improved the patients' function of daily living (but not their cognitive function) at 12 months and had a cost-saving effect, thus determining a yearly net monetary benefit per patient (in terms of lower long-term care costs) of $1,605; the probability of the net monetary benefit being beyond zero was around 91%. However, they concluded that a RCT was required to verify these findings.

Mervin et al. [120] assessed the cost-effectiveness of regular use of a therapeutic pet-type robot (PARO) compared with a normal plush toy and usual care for reducing agitation and medication use in institutionalised dementia patients. The residential facilities were randomised to one of three groups: PARO (individual, non-facilitated 15-minute sessions on three afternoons per week for 10 weeks), plush toy (as per PARO, but with artificial intelligence disabled), and usual care. The incremental cost-effectiveness was measured as the incremental cost per unit improvement in agitation between the two comparative groups (PARO or plush toy vs usual care). The study could not find evidence of a substantial cost-effectiveness for the use of the robotic plushie compared with usual care: the incremental cost per unit improvement in the Cohen-Mansfield Agitation Inventory-Short Form (CMAI-SF) was AU$13.01 for the PARO group and AU$12.85 for the plush toy group. The authors concluded that an inexpensive plush toy may offer greater value for money than the PARO in terms of improving agitation specifically, with an incremental cost per unit in the CMAI-SF of AU$2.17. However, they underline that these costs are also much lower than values estimated for other psychosocial group activities (from approximately AU$280 to AU$6,030) and sensory interventions (from approximately AU$42 to AU$248), suggesting that both a plush toy and the PARO may be cost-effective psychosocial treatment options for agitation.

D'Amico et al. [111] analysed a maintenance CST intervention added to usual care and directed at people with mild to moderate dementia in nine care homes and nine community centres. They performed both cost-effectiveness and cost-utility analyses within a multicentre, single-blind, pragmatic RCT with a subgroup analysis for people taking acetylcholinesterase inhibitors. Participants received the treatment for seven weeks and were randomised to either the weekly maintenance of cognitive stimulation programme added to usual care or usual care alone for 24 weeks. The authors found that the intervention was cost-effective in terms of increased QoL, which was measured using proxy EQ-5D ratings; the mean ICER was about 26,835 per QALY,

and the probability that the intervention would be cost-effective was 54% at the NICE's specified threshold of £30,000/QALY [126]. The intervention was also cost-effective in terms of reducing cognitive impairment as measured with MMSE (Mini Mental State Examination) scores, but not when measured with the Alzheimer's Disease Assessment Scale-Cognitive (ADAS-Cog). Moreover, the authors noted that the intervention, in combination with acetylcholinesterase inhibitors, was more cost-effective than using inhibitors alone on top of usual care.

Orgeta et al. [112] performed a clinical effectiveness and cost-effectiveness pragmatic, multicentre, RCT of a home-based, individual CST intervention for PwDs and their family carers, which was then compared with treatment as usual. The intervention consisted of structured cognitive stimulation sessions for PwDs completed up to three times weekly over 25 weeks. Family caregivers were supported to deliver the sessions at home. Primary outcomes were cognition and QoL for the PwDs, and mental/physical health (measured with the Short Form questionnaire with 12 items) for the family carers. Carers' utility scores were calculated from the EQ-5D by applying published societal weights. They found limited evidence of cost-effectiveness mostly in terms of caregiver's health-related QoL: the incremental health and social care cost per caregiver's QALY gained was approximately £3,100; the probability of the intervention being cost-effective at a WTP per QALY of approximately £30,000 was 81% from the health and social care perspective and 93% from the societal perspective.

Through a consideration of cognition (measured using the MMSE) as the primary outcome and the patient's QoL (using Qualify of Life in Alzheimer's Disease scale [QoL-AD]) as the secondary outcome, Knapp et al. [124] analysed the cost-effectiveness of an evidence-based CST delivered to dementia patients in nursing homes and day centres. The Cochrane systematic reviews on reality orientation and reminiscence therapy for dementia were used to develop a group programme of evidence-based CST. Despite the short follow-up period (eight weeks), the intervention was found to be potentially cost-effective with regard to both outcome measures.

Physical activity

D'Amico et al. [125] examined the cost-effectiveness of a physical activity intervention—a regular and individually tailored walking regimen—directed at community-dwelling patient-caregiver dyads. The exercise

intervention was significantly more cost-effective than treatment, as usual, from both the societal and health and social care perspectives in terms of improvements in behavioural and psychological symptoms (from a societal perspective, the ICER was £421 per incremental difference in the NPI score), while it did not appear to be cost-effective when considering QALY gains (the ICER per QALY calculated using the DEMQOL-Proxy scores and societal weights was £286,440). The authors observed that there was no established cost-effectiveness benchmark for the NPI with which to compare their estimates, while the estimated mean cost per QALY was rather high relative to the £30,000 upper threshold generally associated with cost-effectiveness judgements by NICE in the UK.

Indirect interventions

In an observational, controlled, cohort study, MacNeil Vroomen et al. [58] developed a preliminary analysis of the cost-effectiveness of two case management models adopted in the Netherlands for people with already diagnosed dementia and their informal caregivers against no access to case management (control group). The two models considered were the Intensive Case Management model (ICMM, in which case managers follow the patients and their families and help them navigate medical and psychosocial services) and the Linkage model (LM, which consists of cooperation between the multiple care providers the patients face in their journey). The economic evaluation related incremental costs to incremental effects regarding neuropsychiatric symptoms (measured with the NPI), the psychological health of the informal caregiver (measured with the General Health Questionnaire [GHQ]), and the QALYs of the PwD and informal caregiver (based on the EuroQoL - 5 Dimensions). They found preliminary evidence of cost-effectiveness for the ICMM compared to the control group (the probability that the ICMM was cost-effective in comparison with the control was 92% at a WTP of €30,000 per QALY) and the LM (the probability that the ICMM was cost-effective in comparison to the LM was 97% at a WTP of €30,000 per QALY), but additional evidence was needed due to the fact that the study was not a RCT.

Michalowsky et al. [59] evaluated a community-based DCM program versus usual care (DelpHi-MV trial). DCM is a model of collaborative care aiming to support patients and their caregivers through coordination and management of treatment and care and consists of a nurse-led in-depth assessment of patients' unmet needs to optimise and individualise dementia treatments. The intervention was delivered in participants' homes by

nurses with dementia-specific qualifications. The analysis was conducted from the public payer perspective (considering only outcomes for PwDs) and based on 444 participants (315 in the intervention group, and 129 in the control group) who completed the baseline and at least one follow-up assessment. The HRQoL of the PwDs was assessed via self-rating measures using the 12-item Short Form Health Survey (SF-12), which is considered suitable for mildly to moderately cognitively impaired PwDs. The responses to the SF-12 were converted to health utilities in order to calculate the QALYs for each patient. In the base-case analysis, compared with usual care, the DCM was associated with higher QALY and lower costs after 24 months. In particular, patients treated with the DCM faced higher costs for medications (i.e., higher prescription rates of anti-dementia drugs and medical aids) but had lower costs in terms of in-hospital treatments, nursing home care, and delayed institutionalisation (the time to institutionalisation was delayed on average seven months in patients who received the DCM). The probability of the DCM being cost-effective was 88% at a WTP of €40,000 per QALY gained, a value higher than the NICE's upper threshold.

A recent follow-up study by Rädke et al. [115] focused on subgroups of participants in the DelpHi-MV trial to assess the differences in cost-effectiveness across the subgroups (if any). They found that patient characteristics significantly affected the cost-effectiveness of the DCM; at a WTP of €40,000 per QALY, the intervention was more likely to be cost-effective for females (a probability of 96% vs 16% for males), patients living alone (96% vs 26% for those not living alone), patients with a moderate-to-severe cognitive impairment (100% vs 3% for low cognitive impairment), patients with functional impairment (97% vs 16% for no functional impairment), and patients with a high comorbidity (96% vs 26% for low comorbidity).

Wimo et al. [65] developed a particular cost-effectiveness analysis (a "cost-effectiveness quotient analysis") for three day care units for PwDs with a daily capacity of about nine dementia sufferers served by two specially trained dementia carers. Fifty-five patients in day care and 45 patients on a waiting list for day care (control group) were examined during 12 months in a prospective open non-randomised concurrent control study. All patients included in the study lived in their homes or at a home for the aged. The costs per patient per day were $55 for the day care group and $63 for the controls. Both groups deteriorated in the used indices of QoL (the Index of Wellbeing [IWB] and the Rosser index) during the study year, but the decline was stronger in the control group. The trend indicated that day care was both cost-saving and had better outcomes on the indices, and some underlying variables for the used indices (the ability to dress and undress, and cheerfulness) also

showed significantly better results in favour of day care. Since the changes between the groups were not significant regarding the cost-effectiveness quotient, the authors could not conclude that day care was more cost-effective than a care organisation without day care. However, for a subgroup of patients with the most distressed psychosocial situations, day care was shown to be cost-effective (i.e., it provided the same utilities [QoL indices] of the alternative option but at a lower cost).

Van de Ven et al. [121] investigated differences in the costs of care between a DCM protocol and usual care in a sample of Dutch nursing homes alongside an 18-month cluster-RCT, in which they studied the effectiveness of DCM on residents and staff outcomes (including 318 residents and 376 nursing staff members). Dementia special care units were randomly assigned to DCM or usual care. Nurses from the intervention care homes received DCM training, attended a DCM organisational briefing day, and conducted the four-month DCM intervention twice during the study. A single DCM cycle consisted of observation, feedback to the staff, and action plans for the residents. The authors measured costs related to health care consumption, falls, and psychotropic drug use at the resident level and absenteeism at the staff level. The authors found the DCM intervention to be cost-neutral compared to usual care. However, certain changes within costs could be observed. In particular, the intervention group showed lower costs associated with outpatient hospital appointments over time than the control group, while the use of antipsychotics decreased in both groups (this could be explained as a result of a steady change in the policy of elderly-care physicians to decrease the prescription rate of inappropriate psychotropic drugs). On the staff level, no significant difference between the intervention and control groups for costs associated with absenteeism was found. The authors concluded that, since several studies provided evidence that DCM has positive effects on resident outcome measures such as depression, agitation, and QoL, considerations other than costs might help to determine whether a nursing home should adopt this method.

Interventions for carers

A community-based adult day care service providing support to informal caregivers of PwDs was evaluated by Gaugler et al. [122]. Caregivers in the treatment condition were recruited from 45 adult day programmes. Both the treatment and control groups of caregivers were administered interviews at three intervals: baseline (T1), three months (T2), and one year (T3). Two longitudinal panels were constructed: a short-term (three

months) panel and a long-term (one year) panel. The considered caregiver outcomes were measured in terms of primary stress (through the 7-item Role Overload Scale [ROS]) and depression (using the 20-item Center for Epidemiological Studies Depression Scale [CES-D]). A variety of costs (e.g., adult day services, formal service use, informal sources of care, and employment changes) were estimated for caregivers. The ICERs were calculated as the cost necessary to alleviate role overload and depression by one unit both in the short and long term. In particular, the authors found that the daily costs necessary to alleviate role overload and depression by one unit among adult day service users were $6.83 and $2.90, respectively, over the short term, while these costs were reduced to $4.51 and $2.20, respectively, over the one-year period. The authors concluded that, with consistent utilisation over time, adult day services appeared to have become more cost-effective for caregivers and their care recipients. In fact, there was evidence that the long-term utilisation of day care could help to lessen the time caregivers spent managing symptoms associated with dementia and allow them to spend more time in work-related activities.

Multicomponent interventions

Wolfs et al. [73] studied an intervention adopted in the Netherlands that consisted of an integrated approach protocol involving the use of a diagnostic research centre for psycho-geriatrics. This centre was designed to enable health professionals to first map the needs of the community-dwelling patient and their caregiver, and then deliver a personalised treatment course consisting of different kinds of activities. They found that the intervention was cost-effective in terms of QoL (the ICER was €1,267 per QALY gained), but not in terms of improvements in clinical measures such as cognitive impairment or behavioural and psychological symptoms.

Interventions without evidence of cost-effectiveness

No or insufficient evidence of cost-effectiveness of SCIs has been found in nine of the analysed studies (see Table 5).

Table 5. Studies that do not find evidence of cost-effectiveness of supportive care interventions.

Study	Type of intervention	Intervention Description and Comparator<br>(Setting)	Country	Type of study and economic evaluation<br>(Time horizon)	Sample size No. PwDs /No. Caregivers (Intervention Group/Control Group)	Perspective	PwD Outcome measures	Caregiver Outcome measures	Incremental Cost-Effectiveness Ratio (ICER) and other cost-effectiveness measures	Quality of the study
Woods et al. 2012 [113]	Cognitive	Reminiscence group therapy vs Usual Care<br>(Community-based: Memory clinics and Community mental health teams for older people)	UK	RCT CEA<br>(10 months)	350 / 350<br>(206 / 144)	•Health and social care system	•Quality of life (QoL-AD, EQ-5D)<br>•Autobiographical memory (AMI (E))<br>•Quality of relationship (QCPR)<br>•Depression and anxiety (CSDD, RAID)<br>•BADLs<br>•Service utilisation (CSRI)	•Mental health (GHQ-28)<br>•Quality of life (EQ-5D)<br>•Quality of relationship (QCPR)<br>•Depression and anxiety (HADS)<br>•Caregiving stress (RSS)<br>•Service utilisation (CSRI)	£2,586 per QoL-AD point	Medium
Khan et al. 2019 [114]	Physical activity	Structured physical exercise (aerobic and resistance training at moderate-to-hard intensity) vs Usual Care<br>(Community-based: home)	UK	RCT CUA<br>(12 months)	494 / 494<br>(329 / 165)	•Health and social care system<br>•Societal	•Cognitive impairment (ADAS-Cog)<br>• ADLs (BADLS)<br>•Quality of life (EQ-5D, QoL-AD)<br>•Behavioral and psychological symptoms (NPI)<br>•Service utilisation (CSRI)<br>•Falls and fractures	•Quality of life (EQ-5D)<br>•Caregiver burden (ZBI)	- £74,227 per QALY gained (patient) (intervention more costly and less effective)	High
Livingston et al. 2019 [61]	Indirect	MARQUE intervention (mandatory training sessions for staff and implement new procedures to reduce agitation) vs Usual Care<br>(Nursing Home)	UK	RCT CUA<br>(8 months)	318 / 354<br>(PwD: 155/163) (Staff: 175/179)	•Health and social care system	•Agitation (CMAI)<br>•Behavioural and psychological symptoms (NPI)<br>•Dementia severity (CDR)<br>• Antipsychotic drug use<br>•Quality of life (DEMQOL, EQ-5D)<br>•Service utilisation (CSRI)	•Caregiver burnout (MBI)<br>•Sense of competence (SCD)<br>•Abusive behaviour by staff (STS)	£14,064 per QALY gained (patient)	Medium
Meeuwsen et al. 2013 [62]	Indirect	Memory clinics (providing drugs and non-pharmacological interventions) vs Care by GP<br>(Community-based: Memory clinics)	Netherlands	RCT CUA<br>(12 months)	160 / 160<br>(83 / 77)	•Societal	•Quality of life (EQ-5D)<br>•ADLs<br>•IADLs<br>•Service utilisation	•Quality of life (EQ-5D)	€41,442 per QALY lost (patient + caregiver)	High
Drummond et al. 1991 [64]	For Caregivers	Caregiver support program (nurse visits, support groups and respite care) vs Usual Care<br>(Community-based: home)	Canada	RCT CUA<br>(6 months)	0 / 42<br>(22 / 20)	•Health and social care system	----	•Depression (CES-D)<br>•Anxiety (STAI)<br>•Quality of life (CQLI)	CA$20,036 per QALY gained	Low

Study	Type of intervention	Intervention Description and Comparator<br><br>(Setting)	Country	Type of study and economic evaluation<br><br>(Time horizon)	**Sample size** No. PwDs/No. Caregivers (Intervention Group/Control Group)	Perspective	PwD Outcome measures	Caregiver Outcome measures	Incremental Cost-Effectiveness Ratio (ICER) and other cost-effectiveness measures	Quality of the study
Joling et al. 2013 [67]	For Caregivers	Family meetings vs Usual Care<br><br>(Community-based: home)	Netherlands	RCT<br>CUA<br><br>(12 months)	192 / 192<br><br>(96 / 96)	•Societal	•Health Related Quality of life (SF-12)<br>•Service utilisation	•Health Related Quality of life (SF-12)<br>•Depression and anxiety (MINI)<br>•Service utilisation	-€807,703 per QALY (dyad),<br>-240,247 per QALY (patient),<br>-€24,472 per QALY (caregiver)<br>(intervention more costly and less effective)	High
Wilson et al. 2009 [123]	For Caregivers	Social care intervention for caregivers (contact with a befriender facilitator)<br>vs Usual Care<br><br>(Community-based: home)	UK	RCT<br>CUA<br><br>(15 months)	0 / 190<br><br>(93 / 97)	•Societal	----	•Depression and anxiety (HADS)<br>•Quality of life (EQ-5D)	£105,954 per QALY (caregiver)	High
Søgaard et al. 2013 [74]	Multicomponent	Psychosocial intervention (DAISY)<br>vs Usual Care<br><br>(Community-based: Primary care and memory clinics)	Denmark	RCT<br>CUA<br><br>(36 months)	330 / 330<br><br>(163 / 167)	•Health and social care system<br>•Societal	•Quality of life (EQ-5D)<br>•Service utilisation<br>•Institutionalisation rates	•Quality of life (EQ-5D)<br>•Time spent caregiving (RUD)	Incremental cost per QALY <0 (patient+carer)<br>(intervention more costly and less effective)	High
Eloniemi-Sulkava et al. 2009 [75]	Multicomponent	Multicomponent support intervention for couples<br>vs Usual Care<br><br>(Community-based)	Finland	RCT<br>CCA<br><br>(2 years)	125 / 125<br><br>(63 / 62)	•Health and social care system	•Comorbidity (CCI)<br>•Physical functioning (Barthel Index)<br>•Behavioral and psychological symptoms (NPI)<br>•Service utilisation<br>•Institutionalisation	•Caregiver burden (ZBI)	€7,985 saved in healthcare services compared to the control group (not considering the intervention costs)	Low

Legend

ADAS-Cog: Alzheimer's Disease Assessment Scale-Cognition subscale; ADLs: Activities of Daily living; AMI (E): Autobiographical memory interview (extended version); BADLS: Bristol Activities of Daily Living Scale; CCA: Cost consequence analysis; CCI: Charlson Comorbidity Index; CDR: Clinical Dementia Rating; CEA: Cost-effectiveness analysis; CES-D: Center for Epidemiologic Studies Depression Scale; CMAI = Cohen- Mansfield Agitation Inventory; CQLI: Caregiver Quality of Life Instrument; CSRI: Client Service Receipt Inventory CSSD: Cornell Scale for Depression in Dementia; CUA: Cost-utility analysis; DEMQOL Proxy: Dementia Quality of Life score reported by a carer; EQ-5D: EuroQol-5 Dimensions; GHQ-28: General Health Questionnaire - 28 item version; HADS: Hospital Anxiety and Depression Scale; IADLs: Instrumental Activities of Daily Living; MBI: Maslach Burnout Inventory; MINI: Mini International Neuropsychiatric Interview; NPI: Neuropsychiatric Inventory; QCPR: Quality of Caregiver–Patient Relationship; QoL-AD: Quality of Life-Alzheimer's Disease scale; RAID: Rating Anxiety in Dementia; RCT: Randomised controlled trial; RSS: Relatives' Stress Scale; RUD: Resource utilization in dementia-instrument; SCD: Sense of Competence in Dementia; SF-12: Short Form questionnaire-12 items; STAI: State-Trait Anxiety Inventory; STS: Staff Tactics Scale; ZBI: Zarit Burden Interview (self-reported questionnaire used to assess carer burden).

Cognitive stimulation

A reminiscence group therapy intervention for community-dwelling dementia patients and their caregivers in the UK was evaluated by Woods et al. [113]. The aim of the study was to assess, through a multicentre, pragmatic RCT, the effectiveness and cost-effectiveness of joint reminiscence groups for PwDs and their family caregivers as compared with usual care. Most participants were recruited through memory clinics and community mental health teams for older people. The analysed intervention consisted of joint reminiscence groups held weekly for 12 consecutive weeks, followed by monthly maintenance sessions for a further seven months. The primary outcome measures were self-reported QoL for the PwD (measured with QoL-AD and EQ-5D scores) and psychological distress for the carer (General Health Questionnaire - 28 item version [GHQ-28]). The study did not provide support for the cost-effectiveness of joint reminiscence groups for PwDs and their carers.

Physical activity

The Dementia and Physical Activity (DAPA) trial aimed at estimating the clinical effectiveness and cost-effectiveness at 12 months of a bespoke exercise programme, in addition to usual care, on the cognitive impairment (primary outcome), function, and QoL of people with mild to moderate dementia (MMD) and their carers' burden and QoL. Generic HRQoL was measured using the EQ-5D, three-level version, while dementia-related QoL was measured using the QoL-AD scale. Khan et al. [114] performed a cost-utility analysis of the intervention, which comprised both aerobic and resistance training programmes targeted at community-dwelling PwDs. The analysis found no impact on patient or caregiver outcomes and no evidence of cost-effectiveness, with a mean ICER of -£74,227 per QALY gained (i.e., the intervention was costlier and less effective than usual care). The probability that the DAPA was cost-effective was lower than 1% across the cost-effectiveness thresholds, and the incremental net monetary benefit ranged from –£2,601 to -£2,158 at cost-effectiveness thresholds between £15,000 and £30,000 per QALY. Therefore, the exercise intervention was dominated by usual practice in health economic terms.

Indirect interventions

Livingston et al. [61] performed a cost-effectiveness and cost-utility analysis of the MARQUE intervention targeted towards the staff of 20 nursing homes in the UK. MARQUE was an evidence-based manualised intervention delivered by supervised graduate psychologists to staff in six interactive training sessions on procedures to reduce agitation in residents with dementia. The authors considered both PwD and formal caregiver outcomes (the primary outcome was residents' agitation score at eight months, which was measured using the CMAI). They found that the intervention was not associated with a significant improvement in agitation or QoL, a reduction in possibly abusive behaviours, or a staff reduction in burnout or improvement in the sense of competence. Moreover, the prescription of antipsychotic drugs was not significantly different between the MARQUE group and the "treatment as usual" group. Their economic analysis showed that the intervention could be cost-effective, since the mean incremental cost per QALY gained of £14,064 was less than the NICE threshold of £20,000, but with a relatively low probability (62%). However, these results were driven by assumptions about the cost of the intervention, and the sensitivity analysis suggested that implementation on the basis of QoL should be interpreted with caution. The authors concluded that the implementation of MARQUE should not be recommended on the basis of differences in costs, QALYs, or cost-effectiveness and suggested that higher intensity interventions would be required for people with agitation in care homes.

Meeuwsen et al. [62] performed a cost-utility analysis on Dutch memory clinics in comparison with care provided by a general practitioner (GP) to community-dwelling patients newly diagnosed with MMD and their informal caregivers, with a 12-month follow-up. Compared to GPs' care, treatment provided by the memory clinics was on average €1,024 cheaper and showed a non-significant decrease of 0.025 QALYs (the ICER's denominator was the sum of the patient and caregiver QALYs). Therefore, they found no evidence that memory clinics were more cost-effective compared to general practitioners with regard to post-diagnosis treatment and coordination of care of patients with dementia in the first year after diagnosis.

Interventions for carers

Drummond et al. [64] performed a cost-utility analysis concurrently with a randomised trial comparing a Caregiver Support Programme (CSP) with existing conventional community nursing care for those caring for

elderly relatives at home. The intervention included nurse visits, respite care, and support group sessions. The differences in resource consumption were compared with changes in caregivers' QoL, as measured by the Caregiver Quality of Life Instrument (CQLI). They found an ICER of CAD$20,000 per caregiver's QALY, but due to the statistically non-significant difference in outcome levels, evidence of cost-effectiveness was considered scarce.

Joling et al. [67] studied the cost-utility of regular family meetings to prevent depression and anxiety in informal caregivers of community-dwelling PwDs. Outcome measures included the QALYs of caregivers and patients and the incidence of depression and anxiety disorders in caregivers. The most important cost drivers involved informal care costs, day treatment, and admission costs of hospital and long-term care facilities for the patients. They observed no significant differences in total costs between both groups, including costs due to caregivers' work absenteeism and healthcare utilisation. They found that the maximum probability that the intervention was considered cost-effective in comparison with usual care reached 40% for the outcome QALY per patient-caregiver dyad and 60% for the caregivers' incidence of depression and/or anxiety disorders regardless of the willingness-to-pay. They concluded that the intervention was not cost-effective compared to usual care.

The Befriending and Costs of Caring trial (BECCA) aimed to establish whether another intervention aimed at caregivers, namely a structured befriending service, improved the QoL of carers of PwDs, and at what cost. Two back-to-back studies within the BECCA trial (Wilson et al. [123] together with Charlesworth et al. [127]) analysed the intervention consisting of regular contact with a befriender facilitator to help caregivers improve their psychological wellbeing. Participants were family carers who were cohabiting with, or providing at least 20 hours' care per week for, a community-dwelling relative with a primary progressive dementia. The point estimate ICER was £105,954 per QALY gained, with only a 42.2% probability of being below £30,000 per QALY gained (including the PwD's QALYs, the ICER was £28,848, with a 51.4% probability of being below £30,000). The analysis concluded that it was unlikely that befriending was a cost-effective intervention from the societal point of view.

Multicomponent interventions

Søgaard et al. [74] evaluated the effectiveness and cost-effectiveness at 36 months of an early psychosocial intervention aimed at patient-caregiver dyads that included different forms of counselling, education, and support (both individual- and group-based). The study was conducted alongside the randomised controlled multicentre trial of the Danish Alzheimer's Intervention Study (DAISY). QALYs were estimated separately for the patient and the caregiver (using the EQ-5D) before aggregation for the main analysis. The authors found no significant difference in both the measured costs and QALYs between the intervention and control groups, although a tendency was noted for psychosocial care leading to cost increases with informal care that was not outweighed by the tendency for cost savings with formal care. The probability of a composite psychosocial intervention being cost-effective from a societal perspective did not exceed 36% for any threshold value, and the alternative scenario analysis showed that the probability of cost-effectiveness increased over the range of the threshold values used if the cost perspective was restricted to formal health care. The authors recommended for practices in similar settings to provide follow-up with referrals to available local support programmes when needed and to restrict large, multifaceted intervention programmes to patients and caregivers with special needs until further evidence for cost-effectiveness emerges.

Eloniemi-Sulkava et al. [75] analysed the cost savings associated with a multicomponent intervention directed at community-dwelling patient-spouse dyads. Intervention couples were provided with a multicomponent intervention program with a family care coordinator, a geriatrician, support groups for caregivers, and individualised services. The considered outcomes were time from enrolment to institutionalisation of spouses with dementia and the use of services and service expenditures of couples. At 1.6 years, two times as many PwDs in the control group were in long-term institutional care as in the intervention group. Over a two-year time horizon (at the end of the intervention), the authors found a substantial equivalence in the institutionalisation risk between the control and the treated groups and a decrease in healthcare costs for the intervention group (the mean difference was €7,985 per capita per year) due to a reduction in the use of community services and expenditures. However, when the intervention costs were included, the differences between the groups were not significant.

Discussion

Main findings

This systematic review highlights the main evidence on the cost-effectiveness of SCIs for PwDs and their caregivers. The analysed studies were quite heterogeneous in quality and included relevant costs and outcome measures. Nevertheless, the higher quality studies may provide useful findings on the value for money of specific SCIs.

Ten studies provided evidence of high cost-effectiveness for seven SCIs (see Table 3): two cognitive stimulation and occupational programmes for community-dwelling PwDs [108, 116]; two indirect interventions (a group living service for PwDs [63] and a home care service [109]); two interventions, START and REACH II, aimed at caregivers of community-dwelling PwDs [66, 68,117-118]; and one multicomponent intervention, WHELD, targeted towards patients in nursing homes [71-72]. Three of these SCIs were found dominant (i.e., less costly and more effective) over usual care, including the community-based occupational therapy intervention from memory clinics analysed by Graff et al. [108], the group living service considered by Wimo et al. [63], and the multicomponent WHELD intervention for resident PwDs [71-72]. START, the individual psychosocial therapy programme aimed at informal caregivers, was found to be more effective and costlier than usual care, but its ICER had a high probability of being within the acceptability threshold range of £20,000–30,000 per QALY currently used by the UK National Institute for Health and Care Excellence (NICE), which is also commonly used as an international benchmark [126]. For the other three SCIs—the cognitive stimulation programme for the patient-caregiver dyad TAP and two other community-based indirect interventions—the ICER was expressed as an incremental cost per unit of effectiveness: for the TAP programme [116] and the psychosocial programme for caregivers REACH II [68] the cost of an additional hour of caregiving time that can be saved by the intervention was well below the opportunity cost of caregiver's time; for the Dutch Geriatric Intervention Programme, consisting of regular nurse visits for community-dwelling PwDs [109], the mean incremental cost per successful treatment (prevented functional decline together with improved wellbeing of PwD) was quite low (about €3,500).

Two out of ten studies providing evidence of high cost-effectiveness—those related to the START programme aimed at informal caregivers [66] and community-based occupational therapy interventions from memory clinics [108]—were assessed as being of high quality according to the CHEC criteria [80]; seven other studies [63, 68, 71-72, 109, 117-118] received a medium-quality appraisal, while only one study [116] was classified as being of low quality.

Fourteen studies found evidence of moderate cost-effectiveness for 13 SCIs (see Table 4), including six cognitive stimulation and rehabilitation programmes for community-dwelling PwDs [110, 112], nursing home residents [119, 120], or PwDs in different settings [111, 124]; one light physical exercise programme [125]; four indirect interventions, including personalised case management [58], DCM [59, 115], adult day care services [65], and DCM in a nursing home setting [121]; one intervention for caregivers [122]; and one community-based multicomponent intervention consisting of an integrated and personalised approach [73].

Nine studies found no or insufficient evidence of cost-effectiveness for nine SCIs (see Table 5), including one cognitive stimulation programme based on reminiscence group therapy [113]; one aerobic exercise and resistance training programme [114]; two indirect interventions, including training sessions on agitation for nursing home staff (MARQUE) [61] and memory clinics [62]; three interventions primarily targeted towards caregivers [64, 67, 123]; and two community-based multicomponent interventions [74, 75].

Our analysis partially confirms some results of previous systematic reviews. For example, the systematic reviews by Knapp et al. [27], Nickel et al. [30], and Clarkson et al. [31] suggested that tailored occupational therapy for community-dwelling patients and caregivers [108, 116] and cognitive and long-term psychological interventions directly delivered to PwDs [111, 124] may be either highly or moderately cost-effective with regard to specific outcomes, while joint reminiscence groups for PwDs and carers [113] were found unlikely to be cost-effective. Previous systematic reviews, including the analysis by Jones et al. [29], provided mixed evidence with regard to interventions aimed directly at informal caregivers, even though in more recent reviews [30, 31], the START intervention [66, 117-118] emerged as potentially cost-effective. Our analysis shows that some forms of psychosocial intervention for informal caregivers are highly cost-effective (e.g., the REACH II and START programmes [66, 68, 117-118]) or moderately cost-effective (e.g., the adult day care service analysed by Gaugler et al. [122]), while other similar interventions have little or no cost-effectiveness (e.g., family meetings and befriending [67, 123]).

In contrast to other reviews, we found a high value for money of structured multicomponent interventions targeted towards patients in nursing homes, which have the potential to draw benefits from the most cost-effective one-dimensional programmes. This was the case for the UK-based Improving Wellbeing and Health for People with Dementia (WHELD) programme for patients in nursing homes, which combines person-centred care, physical exercise, psychosocial activities, training for care staff, and the development of a system for triggering the appropriate review of antipsychotic medications for PwDs [71,72]. On the contrary, other multicomponent interventions for community-dwelling patients and caregivers were assessed as moderately cost-effective [73] or without any significant cost-effectiveness [74, 75]. We also found evidence of moderate cost-effectiveness of specific cognitive stimulation programmes for institutionalised PwDs (in particular, the learning therapy programme analysed by Sado et al. [119]) or for community-dwelling PwDs and their carers (specifically, the tailored cognitive rehabilitation programme investigated by Clare et al. within the GREAT trial [110]). In contrast to previous reviews [30, 31], we were not able to find clear evidence of value for money for exercise programmes. For example, the individually tailored exercise intervention considered by D'Amico et al. [125] significantly improved patients' NPI scores but, owing to its high costs, it did not appear cost-effective when considering QALY gains; the DAPA, another exercise programme that was studied by Khan et al. [114], was dominated by usual practice in terms of cost-effectiveness. Similarly, several indirect interventions centred around organisational and environmental changes showed either moderate [58-59, 65, 121] or no [61-62] cost-effectiveness.

Methodological and operational challenges for the cost-effectiveness of supportive care interventions

From this review, we can identify a number of critical issues concerning both the methodology of economic evaluations and actual barriers to achieving better value for money of SCIs for dementia care.

A first issue is the high methodological heterogeneity of the available studies in terms of quality, populations studied (regarding severity, comorbidity, and care settings), and the inclusion of relevant costs and outcome measures, which make it difficult to generalise their results. A frequent limitation of the analysed studies is the

short time frame adopted for measuring most outcomes and costs. This is a result of the fact that most studies were trial-based evaluations. Additional research would be required to investigate the SCIs' effects over longer time horizons. For example, using a decision analysis modelling strategy to compare the costs and effectiveness of the interventions in the longer term could be an option to explore.

A second methodological issue concerns the instruments used to measure the outcomes of SCIs in terms of QoL for PwDs and to derive QALYs in cost-utility analyses. The reviewed studies applied two types of instruments: a) generic instruments to assess HRQoL, such as the EQ-5D [58, 61-62, 73-74, 111, 113-114, 119, 123], the IWB scale [63, 65], the SF-12 [59, 67], and the WHOQoL-BREF [110]; and b) dementia-specific instruments to measure the QoL of PwDs, such as the QoL-AD [111-114, 123-124], the DEMQOL [110-111], and the DEMQOL-Proxy [61, 71-72, 111-112, 125]. The generic and dementia-specific QoL measures generally did not provide consistent cost-effectiveness findings. In particular, generic measures may not capture all relevant aspects associated with PwDs' experiences, even though instruments such as the SF-12 or EQ-5D have been shown to be suitable for HRQoL self-reporting by PwDs in mild and moderate stages [59, 62, 111, 113-114, 123].

Since the assessment of self-report HRQoL and dementia-related QoL in PwDs is often characterised by recall bias and missing values, many studies opted for using carer-proxy reports of PwD QoL [58, 61, 67, 71-74, 111-114, 117,125]; another option was to convert data from clinical/health measures into QoL estimates according to the views of expert external observers [63, 65]. However, there are important differences between self-report and carer-proxy (or expert-proxy) reports [30,128-129]. For this reason, it may be useful to use both self and proxy ratings in the economic analyses of SCIs. Actually, some analysed studies [111-114] provided both measures of PwDs' QoL, thus confirming that self-rated and proxy QoL measures often have low levels of overall agreement and therefore cannot be assumed to substitute for each other. Furthermore, because the outcomes for caregivers and PwDs are typically interlinked, it is important to jointly assess the QoL of patient-caregiver dyads in order to take into account the type of caregiving relationship, which is an area of outcome assessment that has not yet been adequately developed. Four studies considered in our review assessed the QoL of patient-caregiver dyads [58, 62, 67, 74] by calculating the combined QALY scores through the simple summing of the QALYs for the PwD and the caregiver.

Another methodological issue concerns the identification and evaluation of the costs of SCIs. In this respect, 18 out of 33 of the studies under review adopted a narrow perspective, looking only at the health and social care system and overlooking the opportunity costs of informal caregiver inputs and the impacts of caring on their own health and wellbeing. Given the pivotal roles of family and other carers in dementia care, several authors [27, 30] recommend that economic evaluations of SCIs for PwDs take on a societal perspective, including all relevant costs irrespective of where they occur and where they are funded.

Regarding the operational challenges surrounding the adoption of cost-effective SCIs, a first issue to consider is that pure cost-effectiveness analyses measure the ICER as an incremental cost per one-point difference in specific outcome measures, such as the MMSE [111, 124], NPI [125], CMAI [72, 120], or carer's HADS scores [66, 117-118]. However, in contrast to a cost-utility analysis, where the ICER is expressed in terms of the incremental cost per QALY gained and the acceptability threshold range of £20,000–30,000 per QALY is frequently used, no established cost-effectiveness benchmark exists for such outcome changes. Therefore, it is quite difficult to ascertain whether a particular SCI represents good value for money to the health and social care system, because we do not know the decision maker's willingness-to-pay for a one-unit reduction in the MMSE NPI, CMAI, or HADS scores.

Another operational challenge underlined by Knapp et al. [27] is that the cost-effectiveness of SCIs for PwDs depends crucially on the degree of integration between health and social care services, which are often delivered by different providers and funded from different budgets. It is therefore crucial to improve coordination between these services in order to increase the efficiency and effectiveness of interventions for PwDs.

Limitations

The high heterogeneity of the analysed studies with regard to the study populations considered, relevant costs and outcome measures, time horizons, and perspectives of the analyses made it impossible to perform a meta-analysis of the studies; therefore, we used a narrative summary approach.

Even though we conducted a comprehensive literature search based on extensive search terms, some papers meeting the criteria for inclusion might not have been identified. Furthermore, our systematic review may be subject to a language bias, as only publications in English or those with an abstract in English were included. Some studies reviewed [63-65] were published years before the development of guidelines for assessing the methodological quality of health economics evaluations, such as the CHEC criteria [80]; therefore, their quality assessment may have been compromised. In any event, we decided to include these studies in the review because they evaluated forms of home support not considered by more recent studies, thereby allowing us to cover a wider range of SCIs for PwDs.

Future research

Our systematic review has highlighted the potential cost-effectiveness of multicomponent SCIs targeted towards patients in nursing homes (e.g., the WHELD programme [71-72]) that combine several interventions (person-centred care, physical exercise, psychosocial activities, behaviour management and training for care staff, the development of multi-disciplinary teams) with positive effects in terms of a decrease in challenging behaviour and the prescription of psychoactive drugs. Other studies [73-75] provided mixed evidence with regard to the value for money of community-based structured multicomponent interventions targeted at persons with MMD. Future research should therefore examine the cost-effectiveness of structured multicomponent interventions in different care settings and consider subgroups of PwDs at different disease stages. Furthermore, it is important to assess the impact on cost-effectiveness of the different components of multi-disciplinary interventions by focusing on the assessment of the roles of care coordination and case management. Eliciting the contribution of each component to the SCIs' costs and outcomes would be important in terms of policy by highlighting how and why specific interventions may work to benefit PwDs and/or their caregivers.

Similar to other systematic reviews [29-31], we found mixed evidence with regard to SCIs targeted towards informal caregivers. Specifically, some forms of psychosocial intervention for informal caregivers are highly cost-effective [66, 68, 117-118] or moderately cost-effective [122], while others have shown little or no cost-

effectiveness [67, 123]. As such, further investigation is needed to ascertain the real effects of interventions aimed directly at carers.

Conclusion

To assess the current state of research on the cost-effectiveness of SCIs for dementia, we performed a systematic review of the economic evidence, which is still scarce despite the several calls for action that have been made in the past few years [12, 27, 29, 130]. We reviewed 33 studies that analysed 29 SCIs located at different stages of the care pathway for dementia that were generally directed at patient-caregiver dyads. Most interventions (23 out of 29) were implemented in European countries with comparable underlying health and social care systems.

We found that the most promising SCIs in terms of cost-effectiveness were some multicomponent interventions targeted towards nursing home residents, such as the WHELD programme [71-72], together with some forms of tailored occupational therapy [108, 116] and home care support services [63, 114] for community-dwelling PwDs. Our analysis has also shown that some forms of psychosocial intervention for informal caregivers of community-dwelling PwDs, such as the REACH II and START programmes [66, 68, 117-118], were highly cost-effective. These results suggest the importance of policies promoting the adoption of effective supportive care interventions to integrate or substitute the use of medications to increase the economic sustainability of dementia care.

Further research is required to establish the cost-effectiveness of structured multicomponent interventions in different care settings by considering subgroups of PwDs at different disease stages and assessing the impact of each component of the intervention. Moreover, since the evidence on the cost-effectiveness of SCIs targeted towards informal caregivers is mixed, further investigation is needed to ascertain the real effects of these interventions on both the PwD and his/her carer.

Supporting Information

Checklist S1. PRISMA Checklist

S1 File. Electronic Search Strategy

S1 Table. Assessment of methodological quality of the studies with evidence of high cost-effectiveness of SCIs

S2 Table. Assessment of methodological quality of the studies with evidence of moderate cost-effectiveness of SCIs

S3 Table. Assessment of methodological quality of the studies without evidence of cost-effectiveness of SCIs

Acknowledgments

We thank Francesco Miele and Federico Neresini for their useful comments. This study has been carried out within the research project "Supportive care for elderly people with severe cognitive impairment" sponsored by the Civitas Vitae Research Centre at Fondazione OIC, Padova, Italy.

References

1. Nichols E, Szoeke CEI, Vollset SE, Abbasi N, Abd-Allah F, Abdela J, et al. Global, regional, and national burden of Alzheimer's disease and other dementias, 1990–2016: a systematic analysis for the Global Burden of Disease Study 2016. The Lancet Neurology [Internet]. 2019 Jan; 18(1):88–106. doi: 10.1016/S1474-4422(18)30403-4

2. Livingston G, Sommerlad A, Orgeta V, Costafreda SG, Huntley J, Ames D, et al. Dementia prevention, intervention, and care. The Lancet [Internet]. 2017 Dec; 390(10113):2673–734. doi: 10.1016/S0140-6736(17)31363-6

3. Samia LW, Aboueissa A-M, Halloran J, Hepburn K. The Maine Savvy Caregiver Project: Translating an Evidence-Based Dementia Family Caregiver Program Within the RE-AIM Framework. Journal of Gerontological Social Work [Internet]. 2014 May 15; 57(6–7):640–61. doi: 10.1080/01634372.2013.859201

4. Black BE, Johnston D, Reuland M, Lyketsos C, Samus QM. P1-549: Unmet Needs of Community-Residing Persons with Dementia. Alzheimer's & Dementia [Internet]. 2006 Jul 1; 14(7S_Part_9):P543–4. doi: 10.1016/j.jalz.2018.06.560

5. Zhu CW, Scarmeas N, Ornstein K, Albert M, Brandt J, Blacker D, et al. Health-care use and cost in dementia caregivers: Longitudinal results from the Predictors Caregiver Study. Alzheimer's & Dementia [Internet]. 2014 Mar 17; 11(4):444–54. doi: 10.1016/j.jalz.2013.12.018

6. Roth DL, Fredman L, Haley WE. Informal Caregiving and Its Impact on Health: A Reappraisal From Population-Based Studies. The Gerontologist [Internet]. 2015 Feb 18; 55(2):309–19. doi: 10.1093/geront/gnu177

7. 2014 Alzheimer's disease facts and figures. Alzheimer's & Dementia [Internet]. 2014 Mar; 10(2):e47–92. doi: 10.1016/j.jalz.2014.02.001

8. Weller J, Budson A. Current understanding of Alzheimer's disease diagnosis and treatment. F1000Res [Internet]. 2018 Jul 31; 7:1161. doi: 10.12688/f1000research.14506.1

9. Grossberg GT, Tong G, Burke AD, Tariot PN. Present Algorithms and Future Treatments for Alzheimer's Disease. Fink A, editor. JAD [Internet]. 2019 Feb 19; 67(4):1157–71. doi: 10.3233/JAD-180903

10. Jobke B, McBride T, Nevin L, Peiperl L, Ross A, Stone C, et al. Setbacks in Alzheimer research demand new strategies, not surrender. PLoS Med [Internet]. 2018 Feb 27; 15(2):e1002518. doi: 10.1371/journal.pmed.1002518

11. Cummings J, Lee G, Ritter A, Sabbagh M, Zhong K. Alzheimer's disease drug development pipeline: 2019. Alzheimer's & Dementia: Translational Research & Clinical Interventions [Internet]. 2019 Jan; 5(1):272–93. doi: 10.1016/j.trci.2019.05.008

12. Banerjee S. The use of antipsychotic medication for people with dementia: Time for action. Report. UK Department of Health; 2009 Nov.

13. Yan J. FDA Extends Black-Box Warning to All Antipsychotics. PN [Internet]. 2008 Jul 18; 43(14):1–27. doi: 10.1176/pn.43.14.0001

14. Alzheimer's Disease International. Dementia: a public health priority. WHO, London; 2012.

15. Carter EA, Purvis L. Reducing Potential Overuse of Dementia Drugs Could Lead to Considerable Savings. AARP Public Policy Institute. 2018 Aug; 1-6.

16. Hughes J. Models of dementia care: Person-centered, palliative and supportive. Alzheimer's Australia; 2013.

17. Hughes J, Lloyd-Williams M, Sachs G, editors. Supportive care for the person with dementia [Internet]. Oxford University Press; 2009. doi: 10.1093/acprof:oso/9780199554133.001.0001

18. Oyebode JR, Parveen S. Psychosocial interventions for people with dementia: An overview and commentary on recent developments. Dementia [Internet]. 2016 Jul 4; 18(1):8–35. doi: 10.1177/1471301216656096

19. Zucchella C, Sinforiani E, Tamburin S, Federico A, Mantovani E, Bernini S, et al. The Multidisciplinary Approach to Alzheimer’s Disease and Dementia. A Narrative Review of Non-Pharmacological Treatment. Front Neurol [Internet]. 2018 Dec 13; 9. doi: 10.3389/fneur.2018.01058

20. Carrieri D, Peccatori FA, Boniolo G. Supporting Supportive Care in Cancer: The ethical importance of promoting a holistic conception of quality of life. Critical Reviews in Oncology/Hematology [Internet]. 2018 Nov; 131:90–5. doi: 10.1016/j.critrevonc.2018.09.002

21. Boland J, Johnson MJ. End-of-life care for non-cancer patients. BMJ Support Palliat Care [Internet]. 2013 Mar; 3(1):2–3. doi: 10.1136/bmjspcare-2013-000446

22. The Lancet Neurology. Antipsychotic drugs for dementia: a balancing act. The Lancet Neurology [Internet]. 2009 Feb; 8(2):125. doi: 10.1016/s1474-4422(09)70001-8

23. Miele F, Neresini F, Boniolo G, Paccagnella O. Supportive care for older people with dementia: socio-organisational implications. Ageing and Society [Internet]. 2020 Jul 23; 1–33. doi: 10.1017/S0144686X20000938

24. Ward-Griffin C. Supportive care to family caregivers is not supportive enough: moving towards an equitable approach to dementia home care. Neurodegenerative Disease Management [Internet]. 2012 Apr; 2(2):173–81. doi: 10.2217/nmt.11.83

25. Addressing Dementia [Internet]. OECD Health Policy Studies. OECD; 2015. doi: 10.1787/9789264231726-en

26. Gauthier S, Cummings J, Ballard C, Brodaty H, Grossberg G, Robert P, et al. Management of behavioral problems in Alzheimer's disease. Int Psychogeriatr [Internet]. 2010 Jan 25; 22(3):346–72. doi: 10.1017/s1041610209991505

27. Knapp M, Iemmi V, Romeo R. Dementia care costs and outcomes: a systematic review. Int J Geriatr Psychiatry [Internet]. 2012 Aug 12; 28(6):551–61. doi: 10.1002/gps.3864

28. Livingston G, Kelly L, Lewis-Holmes E, Baio G, Morris S, Patel N, et al. A systematic review of the clinical effectiveness and cost-effectiveness of sensory, psychological and behavioural interventions for managing agitation in older adults with dementia. Health Technology Assessment [Internet]. 2014 Jun; 18(39). doi: 10.3310/hta18390

29. Jones C, Edwards RT, Hounsome B. A systematic review of the cost-effectiveness of interventions for supporting informal caregivers of people with dementia residing in the community. Int Psychogeriatr [Internet]. 2011 Aug 4; 24(1):6–18. doi: 10.1017/S1041610211001207

30. Nickel F, Barth J, Kolominsky-Rabas PL. Health economic evaluations of non-pharmacological interventions for persons with dementia and their informal caregivers: a systematic review. BMC Geriatr [Internet]. 2018 Mar 9; 18(1). doi: 10.1186/s12877-018-0751-1

31. Clarkson P, Davies L, Jasper R, Loynes N, Challis D. A Systematic Review of the Economic Evidence for Home Support Interventions in Dementia. Value in Health [Internet]. 2017 Sep; 20(8):1198–209. doi: 10.1016/j.jval.2017.04.004

32. Wimo A, Guerchet M, Ali G-C, Wu Y-T, Prina AM, Winblad B, et al. The worldwide costs of dementia 2015 and comparisons with 2010. Alzheimer's & Dementia [Internet]. 2016 Aug 29; 13(1):1–7. doi: 10.1016/j.jalz.2016.07.150

33. El-Hayek YH, Wiley RE, Khoury CP, Daya RP, Ballard C, Evans AR, et al. Tip of the Iceberg: Assessing the Global Socioeconomic Costs of Alzheimer's Disease and Related Dementias and Strategic Implications for Stakeholders. JAD [Internet]. 2019 Jul 23; 70(2):323–41. doi: 10.3233/JAD-190426

34. Kingston A, Wohland P, Wittenberg R, Robinson L, Brayne C, Matthews FE, et al. Is late-life dependency increasing or not? A comparison of the Cognitive Function and Ageing Studies (CFAS). The Lancet [Internet]. 2017 Oct; 390(10103):1676–84. doi: 10.1016/s0140-6736(17)31575-1

35. Lewis FI, Torgerson PR. The current and future burden of late-onset dementia in the United Kingdom: Estimates and interventions. Alzheimer’s & Dementia [Internet]. 2016 May 6; 13(1):38–44. doi: 10.1016/j.jalz.2016.03.013

36. Versijpt J. Effectiveness and Cost-Effectiveness of the Pharmacological Treatment of Alzheimer’s Disease and Vascular Dementia. JAD [Internet]. 2014 Sep 2; 42(s3):S19–25. doi: 10.3233/JAD-132639

37. Knapp M, Park A, Burns A. Medications for treating people with dementia: summary of evidence on cost-effectiveness [v4]. PSSRU, London School of Economics and Political Science; 2017 Jul 23.

38. Ballard C, Hanney ML, Theodoulou M, Douglas S, McShane R, Kossakowski K, et al. The dementia antipsychotic withdrawal trial (DART-AD): long-term follow-up of a randomised placebo-controlled trial. The Lancet Neurology [Internet]. 2009 Feb; 8(2):151–7. doi: 10.1016/s1474-4422(08)70295-3

39. Cammisuli DM, Danti S, Bosinelli F, Cipriani G. Non-pharmacological interventions for people with Alzheimer’s Disease: A critical review of the scientific literature from the last ten years. European Geriatric Medicine [Internet]. 2016 Feb; 7(1):57–64. doi: 10.1016/j.eurger.2016.01.002

40. D’Onofrio G, Sancarlo D, Seripa D, Ricciardi F, Giuliani F, Panza F, et al. Non-Pharmacological Approaches in the Treatment of Dementia. In: Update on Dementia [Internet]. InTech; 2016. doi: 10.5772/64232

41. Prince M, Bryce R, Ferri C. World Alzheimer Report 2011: The benefits of early diagnosis and intervention. Alzheimer's Disease International, London; 2011.

42. Breuil V, De Rotrou J, Forette F, Tortrat D, Ganansia-Ganem A, Frambourt A, et al. Cognitive stimulation of patients with dementia: Preliminary results. Int J Geriat Psychiatry [Internet]. 1994 Mar; 9(3):211–7. doi: 10.1002/gps.930090306

43. Spector A, Thorgrimsen L, Woods B, Royan L, Davies S, Butterworth M, et al. Efficacy of an evidence-based cognitive stimulation therapy programme for people with dementia. Br J Psychiatry [Internet]. 2003 Sep; 183(3):248–54. doi: 10.1192/bjp.183.3.248

44. Pimouguet C, Sitta R, Wittwer J, Hayes N, Petit-Monéger A, Dartigues J-F, et al. Maintenance of occupational therapy (OT) for dementia: protocol of a multi-center, randomized controlled and pragmatic trial. BMC Geriatr [Internet]. 2019 Feb 6; 19(1). doi: 10.1186/s12877-019-1046-x

45. Zanetti O. Predictors of cognitive improvement after reality orientation in Alzheimer's disease. Age and Ageing [Internet]. 2002 May 1; 31(3):193–6. doi: 10.1093/ageing/31.3.193

46. Onder G, Zanetti O, Giacobini E, Frisoni GB, Bartorelli L, Carbone G, et al. Reality orientation therapy combined with cholinesterase inhibitors in Alzheimer's disease: randomised controlled trial. Br J Psychiatry [Internet]. 2005 Nov; 187(5):450–5. doi: 10.1192/bjp.187.5.450

47. Woods B, O'Philbin L, Farrell EM, Spector AE, Orrell M. Reminiscence therapy for dementia. Cochrane Database of Systematic Reviews [Internet]. 2018 Mar 1; doi: 10.1002/14651858.cd001120.pub3

48. Kawashima R, Okita K, Yamazaki R, Tajima N, Yoshida H, Taira M, et al. Reading Aloud and Arithmetic Calculation Improve Frontal Function of People With Dementia. The Journals of Gerontology Series A: Biological Sciences and Medical Sciences [Internet]. 2005 Mar 1; 60(3):380–4. doi: 10.1093/gerona/60.3.380

49. Killick J, Allan K. The arts in dementia care: tapping a rich resource. J Dement Care. 1999; 7:35–38.

50. Raglio A, Filippi S, Leonardelli L, Trentini E, Bellandi D. The Global Music Approach to Dementia (GMA-D): evidences from a case report. Aging Clin Exp Res [Internet]. 2018 Feb 20; 30(12):1533–6. doi: 10.1007/s40520-018-0919-8

51. Galbraith B, Larkin H, Moorhouse A, Oomen T. Intergenerational Programs for Persons With Dementia: A Scoping Review. Journal of Gerontological Social Work [Internet]. 2015 Mar 6; 58(4):357–78. doi: 10.1080/01634372.2015.1008166

52. Mitchell G. Use of doll therapy for people with dementia: an overview. Nursing Older People [Internet]. 2014 Apr 30; 26(4):24–6. doi: 10.7748/nop2014.04.26.4.24.e568

53. Zafra-Tanaka JH, Pacheco-Barrios K, Tellez WA, Taype-Rondan A. Effects of dog-assisted therapy in adults with dementia: a systematic review and meta-analysis. BMC Psychiatry [Internet]. 2019 Jan 24; 19(1). doi:.1186/s12888-018-2009-z

54. Douglas S, James I, Ballard C. Non-pharmacological interventions in dementia. Adv psychiatr treat [Internet]. 2004 May; 10(3):171–7. doi: 10.1192/apt.10.3.171

55. King AC. Moderate-Intensity Exercise and Self-rated Quality of Sleep in Older Adults. JAMA [Internet]. 1997 Jan 1; 277(1):32. doi: http://dx.doi.org/10.1001/jama.1997.03540250040029

56. Young A, Dinan S. ABC of Sports Medicine: Fitness for older people. BMJ [Internet]. 1994 Jul 30; 309(6950):331–4. doi: 10.1136/bmj.309.6950.331

57. Somme D, Trouve H, Dramé M, Gagnon D, Couturier Y, Saint-Jean O. Analysis of case management programs for patients with dementia: A systematic review. Alzheimer's & Dementia [Internet]. 2012 Jan 30; 8(5):426–36. doi: 10.1016/j.jalz.2011.06.004

58. MacNeil Vroomen J, Bosmans JE, Eekhout I, Joling KJ, van Mierlo LD, Meiland FJM, et al. The Cost-Effectiveness of Two Forms of Case Management Compared to a Control Group for Persons with Dementia and Their Informal Caregivers from a Societal Perspective. Mihalopoulos C, editor. PLoS ONE [Internet]. 2016 Sep 21; 11(9):e0160908. doi: 10.1371/journal.pone.0160908

59. Michalowsky B, Xie F, Eichler T, Hertel J, Kaczynski A, Kilimann I, et al. Cost-effectiveness of a collaborative dementia care management-Results of a cluster-randomized controlled trial. Alzheimer's & Dementia [Internet]. 2019 Aug 10; 15(10):1296–308. doi: 10.1016/j.jalz.2019.05.008

60. Williams J, Rees J. The use of 'dementia care mapping' as a method of evaluating care received by patients with dementia – an initiative to initiative to improve quality of life. Journal of Advanced Nursing [Internet]. 1997 Feb; 25(2):316–23. doi: 10.1046/j.1365-2648.1997.1997025316.x

61. Livingston G, Barber J, Marston L, Stringer A, Panca M, Hunter R, et al. Clinical and cost-effectiveness of the Managing Agitation and Raising Quality of Life (MARQUE) intervention for agitation in people with dementia in care homes: a single-blind, cluster-randomised controlled trial. The Lancet Psychiatry [Internet]. 2019 Apr; 6(4):293–304. doi: 10.1016/s2215-0366(19)30045-8

62. Meeuwsen E, Melis R, van der Aa G, Golüke-Willemse G, de Leest B, van Raak F, et al. Cost-Effectiveness of One Year Dementia Follow-Up Care by Memory Clinics or General Practitioners: Economic Evaluation of a Randomised Controlled Trial. Meara JG, editor. PLoS ONE [Internet]. 2013 Nov 25; 8(11):e79797. doi: 10.1371/journal.pone.0079797

63. Wimo A, Mattson B, Krakau I, Eriksson T, Nelvig A, Karlsson G. Cost-Utility Analysis of Group Living in Dementia Care. Int J Technol Assess Health Care [Internet]. 1995; 11(1):49–65. doi: 10.1017/s0266462300005250

64. Drummond MF, Mohide EA, Tew M, Streiner DL, Pringle DM, Gilbert JR. Economic Evaluation of A Support Program for Caregivers of Demented Elderly. Int J Technol Assess Health Care [Internet]. 1991; 7(2):209–19. doi: 10.1017/s0266462300005109

65. Wimo A, Mattsson B, Krakau I, Eriksson T, Nelvig A. Cost-effectiveness analysis of day care for patients with dementia disorders. Health Econ [Internet]. 1994 Nov; 3(6):395–404. doi: 10.1002/hec.4730030607

66. Knapp M, King D, Romeo R, Schehl B, Barber J, Griffin M, et al. Cost effectiveness of a manual based coping strategy programme in promoting the mental health of family carers of people with dementia (the START (STrAtegies for RelaTives) study): a pragmatic randomised controlled trial. BMJ [Internet]. 2013 Oct 25; 347(oct25 2):f6342–f6342. doi: 10.1136/bmj.f6342

67. Joling KJ, Bosmans JE, van Marwijk HW, van der Horst HE, Scheltens P, Vroomen JL, et al. The cost-effectiveness of a family meetings intervention to prevent depression and anxiety in family caregivers of patients with dementia: a randomized trial. Trials [Internet]. 2013; 14(1):305. doi: 10.1186/1745-6215-14-305

68. Nichols LO, Chang C, Lummus A, Burns R, Martindale-Adams J, Graney MJ, et al. The Cost-Effectiveness of a Behavior Intervention with Caregivers of Patients with Alzheimer's Disease. Journal of the American Geriatrics Society [Internet]. 2008 Mar; 56(3):413–20. doi: 10.1111/j.1532-5415.2007.01569.x

69. Miles L, McCausland BMS, Patel HP, Amin J, Osman-Hicks VC. A systematic review of the provision and efficacy of patient and carer information and support (PCIS) interventions for patients with dementia and their informal carers. Aging Clin Exp Res [Internet]. 2019 Dec 5; 32(12):2439–48. doi: 10.1007/s40520-019-01428-8

70. Merlo P, Devita M, Mandelli A, Rusconi ML, Taddeucci R, Terzi A, et al. Alzheimer Café: an approach focused on Alzheimer's patients but with remarkable values on the quality of life of their caregivers. Aging Clin Exp Res [Internet]. 2017 Oct 11; 30(7):767–74. doi: 10.1007/s40520-017-0844-2

71. Ballard C, Corbett A, Orrell M, Williams G, Moniz-Cook E, Romeo R, et al. Impact of person-centred care training and person-centred activities on quality of life, agitation, and antipsychotic use in people with dementia living in nursing homes: A cluster-randomised controlled trial. Miller BL, editor. PLoS Med [Internet]. 2018 Feb 6; 15(2):e1002500. doi: 10.1371/journal.pmed.1002500

72. Romeo R, Zala D, Knapp M, Orrell M, Fossey J, Ballard C. Improving the quality of life of care home residents with dementia: Cost-effectiveness of an optimized intervention for residents with clinically significant agitation in dementia. Alzheimer's & Dementia [Internet]. 2018 Nov 20; 15(2):282–91. doi: 10.1016/j.jalz.2018.08.010

73. Wolfs CAG, Dirksen CD, Severens JL, Kessels A, Verkaaik M, Verhey FRJ. De kosten en opbrengsten van een geïntegreerde aanpak bij dementie. Tijdschrift voor Psychiatrie. 2011; 53:657-665.

74. Søgaard R, Sørensen J, Waldorff FB, Eckermann A, Buss DV, Phung KTT, et al. Early psychosocial intervention in Alzheimer's disease: cost utility evaluation alongside the Danish Alzheimer's Intervention Study (DAISY). BMJ Open [Internet]. 2014 Jan; 4(1):e004105. doi: 10.1136/bmjopen-2013-004105

75. Eloniemi-Sulkava U, Saarenheimo M, Laakkonen M-L, Pietilä M, Savikko N, Kautiainen H, et al. Family Care as Collaboration: Effectiveness of a Multicomponent Support Program for Elderly Couples with Dementia. Randomized Controlled Intervention Study. Journal of the American Geriatrics Society [Internet]. 2009 Dec; 57(12):2200–8. doi: 10.1111/j.1532-5415.2009.02564.x

76. Wright J, Foster A, Cooper C, Sprange K, Walters S, Berry K, et al. Study protocol for a randomised controlled trial assessing the clinical and cost-effectiveness of the Journeying through Dementia (JtD) intervention compared to usual care. BMJ Open [Internet]. 2019 Sep; 9(9):e029207. doi: 10.1136/bmjopen-2019-029207

77. Drummond MF, Sculpher MJ, Claxton K, Stoddart GL, Torrance GW. Methods for the Economic Evaluation of Health Care Programmes. Fourth Edition. Oxford University Press, London; 2015.

78. Preferred reporting items for systematic review and meta-analysis protocols (PRISMA-P) 2015: elaboration and explanation. BMJ [Internet]. 2016 Jul 21; i4086. doi: 10.1136/bmj.i4086

79. Tacconelli E. Systematic reviews: CRD's guidance for undertaking reviews in health care. The Lancet Infectious Diseases [Internet]. 2010 Apr; 10(4):226. doi: 10.1016/s1473-3099(10)70065-7

80. Evers S, Goossens M, de Vet H, van Tulder M, Ament A. Criteria list for assessment of methodological quality of economic evaluations: Consensus on Health Economic Criteria. Int J Technol Assess Health Care [Internet]. 2005 Apr; 21(2):240–5. doi: 10.1017/s0266462305050324

81. Landeiro F, Walsh K, Ghinai I, Mughal S, Nye E, Wace H, et al. Measuring quality of life of people with predementia and dementia and their caregivers: a systematic review protocol. BMJ Open [Internet]. 2018 Mar; 8(3):e019082. doi: 10.1136/bmjopen-2017-019082

82. Larson EB, Kukull WA, Katzman RL. Cognitive Impairment: Dementia and Alzheimer's Disease. Annu Rev Public Health [Internet]. 1992 May; 13(1):431–49. doi: 10.1146/annurev.pu.13.050192.002243

83. Olde Rikkert MGM, Tona KD, Janssen L, Burns A, Lobo A, Robert P, et al. Validity, Reliability, and Feasibility of Clinical Staging Scales in Dementia. Am J Alzheimers Dis Other Demen [Internet]. 2011 Aug; 26(5):357–65. doi: 10.1177/1533317511418954

84. Cerejeira J, Lagarto L, Mukaetova-Ladinska EB. Behavioral and Psychological Symptoms of Dementia. Front Neur [Internet]. 2012; 3. doi: 10.3389/fneur.2012.00073

85. Bunn F, Burn A-M, Goodman C, Rait G, Norton S, Robinson L, et al. Comorbidity and dementia: a scoping review of the literature. BMC Med [Internet]. 2014 Oct 31; 12(1). doi: 10.1186/s12916-014-0192-4

86. Pivi GAK, Bertolucci PHF, Schultz RR. Nutrition in Severe Dementia. Current Gerontology and Geriatrics Research [Internet]. 2012; 2012:1–7. doi: 10.1155/2012/983056

87. Regan M. The interface between dementia and mental health: an evidence review. Mental Health Foundation, London; 2016.

88. Ijaopo EO. Dementia-related agitation: a review of non-pharmacological interventions and analysis of risks and benefits of pharmacotherapy. Transl Psychiatry [Internet]. 2017 Oct; 7(10):e1250–e1250. doi: 10.1038/tp.2017.199

89. Millán-Calenti JC, Tubío J, Pita-Fernández S, González-Abraldes I, Lorenzo T, Fernández-Arruty T, et al. Prevalence of functional disability in activities of daily living (ADL), instrumental activities of daily living (IADL) and associated factors, as predictors of morbidity and mortality. Archives of Gerontology and Geriatrics [Internet]. 2010 May; 50(3):306–10. doi: 10.1016/j.archger.2009.04.017

90. Bennett HQ, Norton S, Bunn F, Robinson L, Rait G, Goodman C, et al. The impact of dementia on service use by individuals with a comorbid health condition: a comparison of two cross-sectional analyses conducted approximately 10 years apart. BMC Med [Internet]. 2018 Jul 24; 16(1). doi: 10.1186/s12916-018-1105-8

91. Eska K, Graessel E, Donath C, Schwarzkopf L, Lauterberg J, Holle R. Predictors of Institutionalization of Dementia Patients in Mild and Moderate Stages: A 4-Year Prospective Analysis. Dement Geriatr Cogn Disord Extra [Internet]. 2013; 3(1):426–45. doi: 10.1159/000355079

92. Belle SH. Enhancing the Quality of Life of Dementia Caregivers from Different Ethnic or Racial Groups. Ann Intern Med [Internet]. 2006 Nov 21; 145(10):727. doi: 10.7326/0003-4819-145-10-200611210-00005

93. Zarit SH, Todd PA, Zarit JM. Subjective Burden of Husbands and Wives as Caregivers: A Longitudinal Study. The Gerontologist [Internet]. 1986 Jun 1; 26(3):260–6. doi: 10.1093/geront/26.3.260

94. Yu H, Wang X, He R, Liang R, Zhou L. Measuring the Caregiver Burden of Caring for Community-Residing People with Alzheimer's Disease. Ginsberg SD, editor. PLoS ONE [Internet]. 2015 Jul 8; 10(7):e0132168. doi: 10.1371/journal.pone.0132168

95. Schepers AK, Orrell M, Shanahan N, Spector A. Sense of Competence in Dementia Care Staff (SCIDS) scale: development, reliability, and validity. Int Psychogeriatr [Internet]. 2012 Feb 20; 24(7):1153–62. doi: 10.1017/s104161021100247x

96. Infurna FJ, Gerstorf D, Zarit SH. Substantial Changes in Mastery Perceptions of Dementia Caregivers With the Placement of a Care Recipient. The Journals of Gerontology Series B: Psychological Sciences and Social Sciences [Internet]. 2012 Sep 5; 68(2):202–14. doi: 10.1093/geronb/gbs063

97. Thies W, Bleiler L. 2012 Alzheimer's disease facts and figures Alzheimer's Association ∗. Alzheimer's & Dementia [Internet]. 2012 Mar; 8(2):131–68. doi: 10.1016/j.jalz.2012.02.001

98. von Känel R, Mills PJ, Mausbach BT, Dimsdale JE, Patterson TL, Ziegler MG, et al. Effect of Alzheimer Caregiving on Circulating Levels of C-Reactive Protein and Other Biomarkers Relevant to Cardiovascular Disease Risk: A Longitudinal Study. Gerontology [Internet]. 2012; 58(4):354–65. doi: 10.1159/000334219

99. Joling KJ, van Hout HPJ, Schellevis FG, van der Horst HE, Scheltens P, Knol DL, et al. Incidence of Depression and Anxiety in the Spouses of Patients With Dementia: A Naturalistic Cohort Study of Recorded Morbidity With a 6-Year Follow-Up. The American Journal of Geriatric Psychiatry [Internet]. 2010 Feb; 18(2):146–53. doi: 10.1097/jgp.0b013e3181bf9f0f

100. Richardson TJ, Lee SJ, Berg-Weger M, Grossberg GT. Caregiver Health: Health of Caregivers of Alzheimer’s and Other Dementia Patients. Curr Psychiatry Rep [Internet]. 2013 May 28; 15(7). doi: 10.1007/s11920-013-0367-2

101. Barker HR, Griffiths P, Mesa-Eguiagaray I, Pickering R, Gould L, Bridges J. Quantity and quality of interaction between staff and older patients in UK hospital wards: A descriptive study. International Journal of Nursing Studies [Internet]. 2016 Oct; 62:100–7. doi: 10.1016/j.ijnurstu.2016.07.018

102. Luppa M, Riedel-Heller SG, Stein J, Leicht H, König H-H, van den Bussche H, et al. Predictors of Institutionalisation in Incident Dementia – Results of the German Study on Ageing, Cognition and Dementia in Primary Care Patients (AgeCoDe Study). Dement Geriatr Cogn Disord [Internet]. 2012; 33(4):282–8. doi: 10.1159/000339729

103. Fauth E, Hess K, Piercy K, Norton M, Corcoran C, Rabins P, et al. Caregivers’ relationship closeness with the person with dementia predicts both positive and negative outcomes for caregivers’ physical health and psychological well-being. Aging & Mental Health [Internet]. 2012 Aug; 16(6):699–711. doi: 10.1080/13607863.2012.678482

104. Snyder CM, Fauth E, Wanzek J, Piercy KW, Norton MC, Corcoran C, et al. Dementia caregivers’ coping strategies and their relationship to health and well-being: the Cache County Study. Aging & Mental Health [Internet]. 2014 Aug 5; 19(5):390–9. doi: 10.1080/13607863.2014.939610

105. Moore MJ, Zhu CW, Clipp EC. Informal Costs of Dementia Care: Estimates From the National Longitudinal Caregiver Study. The Journals of Gerontology Series B: Psychological Sciences and Social Sciences [Internet]. 2001 Jul 1; 56(4):S219–28. doi: 10.1093/geronb/56.4.s219

106. Martindale-Adams J, Nichols LO, Zuber J, Burns R, Graney MJ. Dementia Caregivers’ Use of Services for Themselves. GERONT [Internet]. 2015 Sep 8; 56(6):1053–61. doi: 10.1093/geront/gnv121

107. Goren A, Montgomery W, Kahle-Wrobleski K, Nakamura T, Ueda K. Impact of caring for persons with Alzheimer’s disease or dementia on caregivers’ health outcomes: findings from a community based survey in Japan. BMC Geriatr [Internet]. 2016 Jun 10; 16(1). doi: 10.1186/s12877-016-0298-y

108. Graff MJL, Adang EMM, Vernooij-Dassen MJM, Dekker J, Jönsson L, Thijssen M, et al. Community occupational therapy for older patients with dementia and their care givers: cost effectiveness study. BMJ [Internet]. 2008 Jan 2; 336(7636):134–8. doi: 10.1136/bmj.39408.481898.be

109. Melis RJF, Adang E, Teerenstra S, van Eijken MIJ, Wimo A, Achterberg T v., et al. Multidimensional Geriatric Assessment: Back to the Future Cost-Effectiveness of a Multidisciplinary Intervention Model for Community-Dwelling Frail Older People. The Journals of Gerontology Series A: Biological Sciences and Medical Sciences [Internet]. 2008 Mar 1; 63(3):275–82. doi: 10.1093/gerona/63.3.275

110. Clare L, Kudlicka A, Oyebode JR, Jones RW, Bayer A, Leroi I, et al. Goal-oriented cognitive rehabilitation for early-stage Alzheimer's and related dementias: the GREAT RCT. Health Technol Assess [Internet]. 2019 Mar; 23(10):1–242. doi: 10.3310/hta23100

111. D'Amico F, Rehill A, Knapp M, Aguirre E, Donovan H, Hoare Z, et al. Maintenance Cognitive Stimulation Therapy: An Economic Evaluation Within a Randomized Controlled Trial. Journal of the American Medical Directors Association [Internet]. 2015 Jan; 16(1):63–70. doi: 10.1016/j.jamda.2014.10.020

112. Orgeta V, Leung P, Yates L, Kang S, Hoare Z, Henderson C, et al. Individual cognitive stimulation therapy for dementia: a clinical effectiveness and cost-effectiveness pragmatic, multicentre, randomised controlled trial. Health Technol Assess [Internet]. 2015 Aug; 19(64):1–108. doi: 10.3310/hta19640

113. Woods R, Bruce E, Edwards R, Elvish R, Hoare Z, Hounsome B, et al. REMCARE: reminiscence groups for people with dementia and their family caregivers – effectiveness and cost-effectiveness pragmatic multicentre randomised trial. Health Technol Assess [Internet]. 2012 Dec; 16(48). doi: 10.3310/hta16480

114. Khan I, Petrou S, Khan K, Mistry D, Lall R, et al. Does Structured Exercise Improve Cognitive Impairment in People with Mild to Moderate Dementia? A Cost-Effectiveness Analysis from a Confirmatory Randomised Controlled Trial: The Dementia and Physical Activity (DAPA) Trial. PharmacoEconomics Open [Internet]. 2018 Sep 11; 3(2):215–27. doi: 10.1007/s41669-018-0097-9

115. Rädke A, Michalowsky B, Thyrian JR, Eichler T, Xie F, Hoffmann W. Who Benefits Most from Collaborative Dementia Care from a Patient and Payer Perspective? A Subgroup Cost-Effectiveness Analysis. JAD [Internet]. 2020 Mar 24; 74(2):449–62. doi: 10.3233/JAD-190578

116. Gitlin LN, Hodgson N, Jutkowitz E, Pizzi L. The Cost-Effectiveness of a Nonpharmacologic Intervention for Individuals With Dementia and Family Caregivers: The Tailored Activity Program. The American Journal of Geriatric Psychiatry [Internet]. 2010 Jun; 18(6):510–9. doi: 10.1097/jgp.0b013e3181c37d13

117. Livingston G, Barber J, Rapaport P, Knapp M, Griffin M, King D, et al. Long-term clinical and cost-effectiveness of psychological intervention for family carers of people with dementia: a single-blind, randomised, controlled trial. The Lancet Psychiatry [Internet]. 2014 Dec; 1(7):539–48. doi: 10.1016/s2215-0366(14)00073-x

118. Livingston G, Manela M, O'Keeffe A, Rapaport P, Cooper C, Knapp M, et al. Clinical effectiveness of the START (STrAtegies for RelaTives) psychological intervention for family carers and the effects on the cost of care for people with dementia: 6-year follow-up of a randomised controlled trial. Br J Psychiatry [Internet]. 2019 Jul 12; 216(1):35–42. doi: 10.1192/bjp.2019.160

119. Sado M, Funaki K, Ninomiya A, Knapp M, Mimura M. Does the Combination of the Cognitive Interventions Improve the Function of Daily Living and Save the Long-Term Care Cost? A Pilot Study of Effectiveness and Cost Saving Analysis of "Learning Therapy" for People with Dementia. Palm R, editor. JAD [Internet]. 2020 Apr 7; 74(3):775–84. doi: 10.3233/JAD-190886

120. Mervin MC, Moyle W, Jones C, Murfield J, Draper B, Beattie E, et al. The Cost-Effectiveness of Using PARO, a Therapeutic Robotic Seal, to Reduce Agitation and Medication Use in Dementia: Findings from a Cluster–Randomized Controlled Trial. Journal of the American Medical Directors Association [Internet]. 2018 Jul; 19(7):619-622.e1. doi: 10.1016/j.jamda.2017.10.008

121. van de Ven G, Draskovic I, van Herpen E, Koopmans RTCM, Donders R, Zuidema SU, et al. The Economics of Dementia-Care Mapping in Nursing Homes: A Cluster-Randomised Controlled Trial. Quinn TJ, editor. PLoS ONE [Internet]. 2014 Jan 28; 9(1):e86662. doi: 10.1371/journal.pone.0086662

122. Gaugler JE, Zarit SH, Townsend A, Parris Stephens M-A, Greene R. Evaluating Community-Based Programs for Dementia Caregivers: The Cost Implications of Adult Day Services. J Appl Gerontol [Internet]. 2003 Feb; 22(1):118–33. doi: 10.1177/0733464802250049

123. Wilson E, Thalanany M, Shepstone L, Charlesworth G, Poland F, Harvey I, et al. Befriending carers of people with dementia: a cost utility analysis. Int J Geriat Psychiatry [Internet]. 2009 Jun; 24(6):610–23. doi: 10.1002/gps.2164

124. Knapp M, Thorgrimsen L, Patel A, Spector A, Hallam A, Woods B, et al. Cognitive stimulation therapy for people with dementia: cost-effectiveness analysis. Br J Psychiatry [Internet]. 2006 Jun; 188(6):574–80. doi: 10.1192/bjp.bp.105.010561

125. D’Amico F, Rehill A, Knapp M, Lowery D, Cerga-Pashoja A, Griffin M, et al. Cost-effectiveness of exercise as a therapy for behavioural and psychological symptoms of dementia within the EVIDEM-E randomised controlled trial. Int J Geriatr Psychiatry [Internet]. 2015 Oct 21; 31(6):656–65. doi: 10.1002/gps.4376

126. Claxton K, Martin S, Soares M, Rice N, Spackman E, Hinde S, et al. Methods for the estimation of the National Institute for Health and Care Excellence cost-effectiveness threshold. Health Technology Assessment [Internet]. 2015 Feb; 19(14):1–504. doi: 10.3310/hta19140

127. Charlesworth G, Shepstone L, Wilson E, Thalanany M, Mugford M, Poland F. Does befriending by trained lay workers improve psychological well-being and quality of life for carers of people with dementia, and at what cost? A randomised controlled trial. Health Technol Assess [Internet]. 2008 Mar; 12(4). doi: 10.3310/hta12040

128. Arons AM, Krabbe PF, Schölzel-Dorenbos CJ, van der Wilt GJ, Rikkert MGO. Quality of life in dementia: a study on proxy bias. BMC Med Res Methodol [Internet]. 2013 Sep 6; 13(1). doi: 10.1186/1471-2288-13-110

129. Kahle-Wrobleski K, Ye W, Henley D, Hake AM, Siemers E, Chen Y, et al. Assessing quality of life in Alzheimer’s disease: Implications for clinical trials. Alzheimer’s & Dementia: Diagnosis, Assessment & Disease Monitoring [Internet]. 2016 Dec 13; 6(1):82–90. doi: 10.1016/j.dadm.2016.11.004

130. Hughes S, Shuman SB, Wiener JM, Gould E. Research on Supportive Approaches for Family and Other Caregivers. Background Paper. Research Summit on Dementia Care. 2017 Mar.

 Click here to access/download;Figure;Fig 1 - PRISMA chart.tif

Figure 1. PRISMA flow chart of the study selection process.

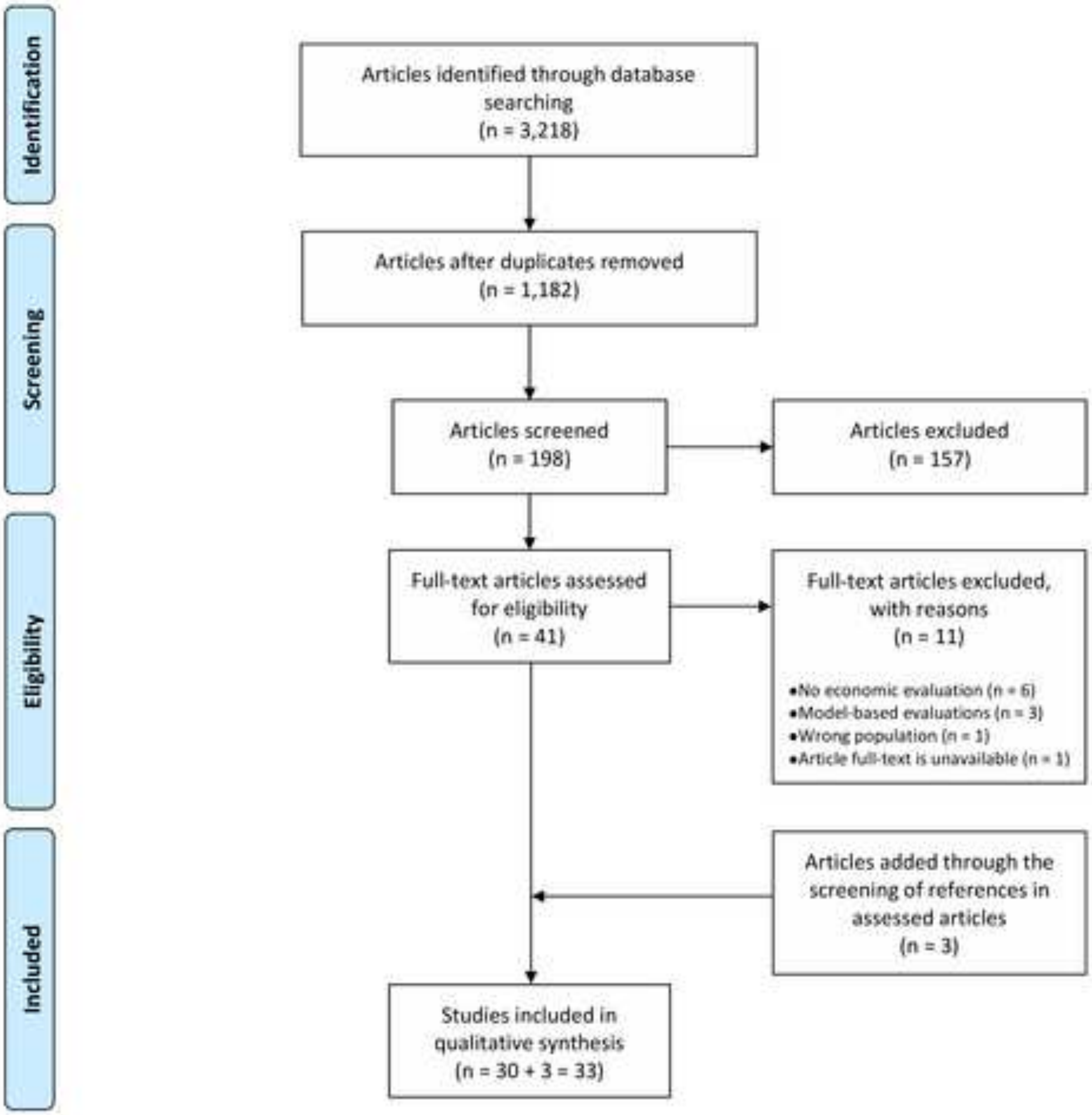

For more information, visit www.prisma-statement.org.

Click here to access/download
Supporting Information
PRISMA checklist S1.docx

Click here to access/download
Supporting Information
S1 File_Electronic Search Strategy.docx

Click here to access/download
Supporting Information
S1 Table.docx

Click here to access/download
Supporting Information
S2 Table.docx

Click here to access/download
Supporting Information
S3 Table .docx
