# Supplementary material for: The value of supportive care: A systematic review of cost-effectiveness of non-pharmacological interventions for dementia
Source: PLoS One. 2023 May 12;18(5):e0285305. doi: 10.1371/journal.pone.0285305 (PMC10180718; doi:10.1371/journal.pone.0285305)
Supplement: Attachment — Submitted filename: Response to Reviewers.docx [file pone.0285305.s011.docx]

**Manuscript No. PONE-D-21-04004R2**

*“The value of supportive care: A systematic review of cost-effectiveness of non-pharmacological interventions for dementia”*

Angelica Guzzon, Vincenzo Rebba*, Omar Paccagnella, Michela Rigon, and Giovanni Boniolo

*Response letter to Editor and reviewers*

Dear Editor,

Thank you very much for your reply and the invitation to revise the manuscript for *PLoS ONE*.

We also would like to thank the reviewers again for spending their time evaluating our manuscript, and for providing valuable feedback that helped to further improve our systematic review. We have been able to incorporate changes to reflect the suggestions provided by the Editor and the reviewers.

As requested, we have corrected and completed the titles of tables 4, 5, 6, 7 and 8 in order to provide a full description of the tables content.

For greater accuracy of information, we also slightly changed the affiliation description of one of the authors, Michela Rigon, and made a small change in the Acknowledgments section.

We think that the new revised version addresses the points raised during the review process.

In this letter we discuss how the revised version has dealt with all suggestions and remarks. We will be glad to provide more details if necessary.

Sincerely,

On behalf of all co-authors,

Vincenzo Rebba

Department of Economics and Management

University of Padova, Italy

**Response to Reviewers**

**Reviewer 1**

The authors did a nice job addressing comments and suggestions. However, I have a few minor additional comments.

Please correct and complete the title of tables as informative format that considered the full description of tables content.

*We thank the Reviewer for providing valuable feedback that really helped to improve our paper. Below is a point-by-point response to the Reviewer’s comments.*

*As requested, we have corrected and completed the titles of Table 4 (on page 21), Table 5 (on page 26), Table 6 (on page 31), Table 7 (on page 38), and Table 8 (on page 44). We made these changes in order to provide a full description of the tables content.*

*We think that the new version addresses the points raised during the review process.*

**Reviewer 2**

The authors did a nice job addressing comments and suggestions. However, I have a few minor additional comments.

Please correct and complete the title of tables as informative format that considered the full description of tables content.

*We thank the Reviewer for providing valuable feedback that really helped to improve our paper. Below is a point-by-point response to the Reviewer’s comments.*

*As requested, we have corrected and completed the titles of Table 4 (on page 21), Table 5 (on page 26), Table 6 (on page 31), Table 7 (on page 38), and Table 8 (on page 44). We made these changes in order to provide a full description of the tables content.*

*We think that the new version addresses the points raised during the review process.*
